# 1,3,4-oxadiazoles as inhibitors of the atypical member of the BET family bromodomain factor 3 from *Trypanosoma cruzi* (*Tc*BDF3)

**DOI:** 10.3389/fmicb.2024.1465672

**Published:** 2024-10-01

**Authors:** Victoria L. Alonso, Andrea M. Escalante, Elvio Rodríguez Araya, Gianfranco Frattini, Luis E. Tavernelli, Diego M. Moreno, Ricardo L. E. Furlan, Esteban Serra

**Affiliations:** ^1^Instituto de Biología Molecular y Celular de Rosario, CONICET-UNR, Rosario, Argentina; ^2^Facultad de Ciencias Bioquímicas y Farmacéuticas, Universidad Nacional de Rosario, Rosario, Argentina; ^3^Instituto de Química Rosario, CONICET-UNR, Rosario, Argentina

**Keywords:** bromodomain, Chagas disease, tubulin acetylation, cytoskeleton, 1,3,4-oxadiazoles, BET bromodomain

## Abstract

Chagas disease, caused by the protozoan parasite *Trypanosoma cruzi*, affects millions globally, with increasing urban cases outside of Latin America. Treatment is based on two compounds, namely, benznidazole (BZ) and nifurtimox, but chronic cases pose several challenges. Targeting lysine acetylation, particularly bromodomain-containing proteins, shows promise as a novel antiparasitic target. Our research focuses on *Tc*BDF3, a cytoplasmic protein, which is crucial for parasite differentiation that recognizes acetylated alpha-tubulin. In our previous study, A1B4 was identified as a high-affinity binder of *Tc*BDF3, showing significant trypanocidal activity with low host toxicity *in vitro*. In this report, the binding of *Tc*BDF3 to A1B4 was validated using differential scanning fluorescence, fluorescence polarization, and molecular modeling, confirming its specific interaction. Additionally, two new 1,3,4-oxadiazoles derived from A1B4 were identified, which exhibited improved trypanocide activity and cytotoxicity profiles. Furthermore, *Tc*BDF3 was classified for the first time as an atypical divergent member of the bromodomain extraterminal family found in protists and plants. These results make *Tc*BDF3 a unique target due to its localization and known functions not shared with higher eukaryotes, which holds promise for Chagas disease treatment.

## 1 Introduction

Chagas disease, caused by the protozoan parasite *Trypanosoma cruzi*, is a neglected disease that affects 6–7 million people worldwide, primarily in the endemic regions of 21 Latin American countries (Pérez-Molina and Molina, 2018). The epidemiological landscape of this disease has been significantly influenced by urbanization, population mobility, and migration patterns, resulting in a shift from rural to urban cases and an increase in occurrences across Canada, the United States, Europe, Africa, the Eastern Mediterranean, and Western Pacific countries. Transplacental and blood-mediated infections are responsible for the current existence of new cases in non-endemic regions. At present, the Chagas disease treatment relies on only two active compounds: nifurtimox and benznidazole, both of which were developed more than five decades ago. These medications are associated with significant side effects. While they are effective during the acute phase of the disease, their efficacy in the chronic phase remains disputed (Chatelain, 2015; Malone et al., 2021). This underscores the urgent need for the development of new, more effective drugs to combat this life-threatening disease.

Lysine acetylation is a reversible posttranslational modification (PTM) observed in various proteins in all cellular compartments. It is involved in multiple processes such as energy metabolism, protein degradation, protein localization, and cell cycle regulation, among others (Ali et al., 2018; Shvedunova and Akhtar, 2022). The dynamics of acetylation as a regulator of protein function depend on the combined actions of writer (acetylase), eraser (deacetylase), and reader (bromodomain and YEATS [Yaf9, ENL, AF9, Taf14, Sas5] domain) proteins of this PTM. Bromodomains (BD) are protein modules of approximately 110 amino acids in length that specifically recognize and bind to acetylated lysines (AcK). These domains feature a left-handed four-*α*-helix bundle structure (αA, αB, αC, and αZ), connected by two loops (ZA and BZ loops), which together form the accessible hydrophobic pocket where AcK recognition occurs (Zeng and Zhou, 2002). Although bromodomain-containing proteins are primarily considered nuclear, there are a limited number of reports in mammals indicating an extranuclear localization (Crowley et al., 2004; Trousdale and Wolgemuth, 2004; Liu et al., 2008).

Our group has been characterizing bromodomain-containing proteins of *T. cruzi* for several years and has identified different BDFs in this parasite (Villanova et al., 2009; Alonso et al., 2014; Ritagliati et al., 2016; Pezza et al., 2022). Surprisingly, *Tc*BDF1 and *Tc*BDF3 were found to be mainly localized in the cytoplasm. *Tc*BDF3, which was found to be associated with acetylated *α*-tubulin at the subpellicular corset and flagella, is necessary for the differentiation of both epimastigotes and intracellular amastigotes to trypomastigotes (Alonso et al., 2014, 2016). This situation appears to be an exclusive characteristic of *T. cruzi* since the BDF3 ortholog in *Trypanosoma brucei* and *Leishmania* spp. has been described as nuclear (Siegel et al., 2009; Schulz et al., 2015; Jones et al., 2022).

The discovery and development of small molecules that can bind bromodomains, blocking their acetyl-lysine binding activity, has experienced enormous growth in the last 15 years. Several of these molecules targeting bromodomains have shown promising activity against various proliferative and proinflammatory pathologies [revised by Gajjela and Zhou (2023)]. According to the Clinical Trials Database from the National Academy of Medicine, there are 57 studies (either completed or still running) with BD inhibitors mainly targeted to different types of cancer and inflammatory diseases.^1^ Among these clinical trials, 47 involve inhibitors of bromodomain and extraterminal (BET) proteins, a sub-family that contains 4 of the 64 bromodomain-containing proteins in humans (Muller et al., 2011; Prinjha et al., 2012).

Parasitic bromodomains have also gained attention as attractive targets for developing new drugs against neglected tropical diseases (Alonso et al., 2019; Tallant et al., 2021). Various BD inhibitors have been tested on protozoa and nematodes (Schulz et al., 2015; Alonso et al., 2016; Chua et al., 2018; García et al., 2018; Ramallo et al., 2018; Chung et al., 2019; Hanquier et al., 2020; Russell et al., 2023; Schiedel et al., 2023; Tavernelli et al., 2024). Additionally, our group described two new chemotypes that bind to *Tc*BDF3 (García et al., 2018; Ramallo et al., 2018). These compounds and previously tested BET bromodomain inhibitor iBET151 showed cytotoxic activity against the different forms of *T. cruzi*. iBET151 has also been proven to bind BDF3 from *T. brucei* (Schulz et al., 2015). Interestingly, iBET151 (and some related compounds) do not kill but induce changes in the transcriptome of *T. brucei*, *similar to* those seen during differentiation from the bloodstream to the procyclic form. The treated parasites experienced changes in surface protein expression, making them more susceptible to the immune system *in vivo* (Schulz et al., 2015; Poli et al., 2023).

One of the compounds previously isolated by our group, A1B4, was identified using a target-directed dynamic combinatorial chemistry (DCC) screening (García et al., 2018). To identify A1B4, a small dynamic library of acyl hydrazones was prepared from aldehydes and acyl hydrazides. *Tc*BDF3 was used as a template to select and amplify suitable binders within the library that were identified using liquid chromatography–mass spectrometry (LC–MS). The most amplified library member, A1B4, showed micromolar-order affinity for *Tc*BDF3, antiparasitic activity against different life-cycle stages in the low micromolar range, and low toxicity against Vero cells. Parasites were rescued from the compound’s toxicity by *Tc*BDF3 overexpression, suggesting that the toxicity of this compound is due to *Tc*BDF3 inhibition (García et al., 2018).

In this report, the binding of A1B4 to *Tc*BDF3 was confirmed using differential scanning fluorescence, fluorescence polarization, and molecular modeling. Also, two novel 1,3,4-oxadiazoles that exhibit improved trypanocidal activity and cytotoxicity profiles were identified. Additionally, through a detailed structural analysis, an extraterminal domain (ET) was discovered at the C-terminal region of *Tc*BDF3. A structure-driven phylogenetic study identified a subfamily of the BET protein family in protists and plants, which includes *Tc*BDF1. This subfamily is characterized by a single bromodomain and an extraterminal (ET) domain, in contrast to the typical 2BD-ET architecture found in metazoans. This finding and the fact that both *Tc*BDF1 and *Tc*BDF3 are essential non-nuclear proteins make them attractive targets for developing new drugs against Chagas disease.

## 2 Materials and methods

### 2.1 Chemistry

#### 2.1.1 Materials and equipment

Proton nuclear magnetic resonance (^1^H-NMR, 300 MHz) and carbon-13 nuclear magnetic resonance (^13^C NMR, 75 MHz) were measured on a Bruker Avance 300 MHz spectrometer. HRMS were obtained using ESI-QTOF Xevo G2S (Waters Corporation). High-performance liquid chromatography-ultraviolet (HPLC-UV) experiments were carried out on an HP 1500 system. HPLC-grade acetonitrile was purchased from Carlo Erba. Milli-Q water was used after filtration through 47-mm nylon membranes with 0.22-μm pore size (Osmonics Filters, Inc.). Trifluoroacetic acid, 4-hydroxy-3-methoxybenzaldehyde (A1), and 2-thiophene-carboxylic acid hydrazide (B4) were purchased from Sigma–Aldrich.

#### 2.1.2 Synthesis procedures

##### 2.1.2.1 Synthesis and characterization of A_1_B_4_

The synthesis of A_1_B_4_ was carried out as previously described (García et al., 2018). In brief, a methanol solution (MeOH) (10 mL) of thiophene-2-carboxylic hydrazide (B4, 5 mmol, 0.71 g) and a MeOH solution (10 mL) of 4-hydroxy-3-methoxybenzaldehyde (A1, 5 mmol, 0.76 g) were mixed with trifluoroacetic acid (TFA) (10 μL). The resulting solution was mixed for 6 h at room temperature. Then, 20 mL of distilled water was added to obtain yellow crystals of A_1_B_4_, which were collected by filtration, washed with cold MeOH, and dried under reduced pressure. Yield 81%, melting point (m.p.) 204°C. ^1^H-NMR (300 MHz, dimethyl sulfoxide [DMSO]-d_6_), *δ* (ppm): 3.84 (3H, s, –CH_3_), 7.05–7.28 (3H, m, Ar–H), 7.30–8.28 (3H, m, thiophene ring-H), 8.31 (1H, s, –CH=N), 9.57 (1H, s, –NH), and 11.69 (1H, s, –OH). ^13^C-NMR (75 MHz, DMSO-d6), δ (ppm): 56.0, 114.7, 116.2, 119.1, 124.0, 128.7, 129.8, 131.3, 137.1, 145.8, 149.1, 149.9, and 168.6. These analyses are consistent with the structure of A1B4 and in agreement with the literature discussed by Modi and Trivedi (2012).

##### 2.1.2.2 Cyclization of A_1_B_4_ using BOC as a protector agent

(*E*)-*N*′-(4-hydroxy-3-methoxybenzylidene)thiophene-2-carbohydrazide (A_1_B_4_, 67.5 mg, 0.24 mmol, 1 eq.) and di-tert-butyl dicarbonate (157 mg, 0.72 mmol, 3 eq.) in MeOH (10 mL) containing triethylamine (Et_3_N) (6 μL) were mixed at 0°C for 1 h. The reaction mixture was left to warm to room temperature and then at 40°C for 3 h. The mixture was concentrated under reduced pressure and the solid residue was purified by flash column chromatography (silica gel 230–400 mesh) eluting with methylene chloride (CH_2_Cl_2_): MeOH (95:5) to obtain (*E*,*Z*)*-*tert-butyl (2*-*methoxy-4-((2-(thiophene-2-carbonyl) hydrazono)methyl)phenyl) carbonate (A_1_B_4_-BOC, 80 mg, 87.5%) as an amorphous white solid. *Rf*: retention factor 0.70 (CH_2_Cl_2_: MeOH = 95:5). ^1^H-NMR (deuterochloroform (CDCl_3_), 300 MHz), *δ* (ppm) 1.57 and 1.59 (s, 9H), 3.91 and 3.94 (s, 3H), 7.12–7.23 (m, 3H), 7.54 (s, 1H), 7.67 (d, *J* = 6 Hz, 1H), 7.87 (br s, 1H), 8.05 (dd, *J =* 6 Hz, 1H), 8.18–8.23 (m, 1H), and 9.85 (br s, 1H). (*E*,*Z*)-tert-butyl (2-methoxy-4-((2-(thiophene-2-carbonyl)hydrazono)methyl)phenyl) carbonate (80 mg, 0.21 mmol, 1 eq.) and chloramine-T (165 mg, 0.59 mmol, 2.8 eq.) were mixed in ethanol (EtOH) (15 mL) at room temperature and then refluxed for 1 h. The mixture was concentrated *in vacuo*. The solid residue was suspended in water (10 mL) and extracted with ethyl acetate (EtOAc) (3 × 5 mL). EtOAc fractions were collected and dried with sodium sulfate (Na_2_SO_4_), and the solvent was evaporated under reduced pressure to obtain a crude mixture. The mixture was further purified by flash column chromatography (silica gel 230–400 mesh) eluting with diethyl ether (EtOEt): EtOAc gradient (from 80:20 to 60:40) to obtain *tert-butyl (2-methoxy-4-(5-(thiophene-2-yl)-1,3,4-oxadiazol-2-yl)phenyl) carbonate* (11.3 mg, 14.3%). *Rf* 0.47 (EtOEt:EtOAc = 6:4). ^1^H-NMR (CDCl_3_, 300 MHz) *δ* (ppm) 1.57 (s, 9H_a_), 3.97 (s, 3H_b_), 7.2 (dd, *J* = 5, 3.8 Hz, 1H_c_), 7.28 (d, *J* = 8.3, 1H_d_), 7.58 (dd, *J* = 5, 1.2 Hz, 1H_e_), 7.67 (dd, *J* = 8.3, 1.9 Hz, 1H_f_), 7.75 (d, *J* = 1.9, 1H_g_), 7.85 (dd, *J* = 3.7, 1.1 Hz, 1H_h_). ^13^C-NMR (CDCl_3_, 75 MHz), *δ* (ppm) 27.6, 56.3, 84.04, 110.9, 119.7, 123.3, 125.1, 128.2, 129.9, 130.3, 143.1, 150.9, 151.9, 161.0, and 163.5. high-resolution mass spectrometry (HRMS) *m*/*z* (ES+) found 375.0993 (100%), C_18_H_18_N_2_O_5_S requires [M + H]^+^ 375.1015. Analytical HPLC-UV (Supplementary Figure S7), rt: retention time 4.04 min, purity >95%.

##### 2.1.2.3 2-methoxy-4-(5-(thiophen-2-yl)-1,3,4-oxadiazol-2-yl)phenol (A_1_B_4c_)

Tert-butyl (2-methoxy-4-(5-(thiophen-2-yl)-1,3,4-oxadiazol-2-yl)phenyl) carbonate (11 mg, 0.03 mmol, 1 eq.) in CH_2_Cl_2_ (1.5 mL) was mixed at 0°C and TFA was slowly added (80 μL, 1.05 mmol, 35 eq.). The reaction mixture was mixed at 0°C for 1 h and then at 40°C for 3.5 h. The reaction mixture was diluted with CH_2_Cl_2_ (5 mL) and washed three times with 3.5 mL of an aqueous sodium bicarbonate (NaHCO_3_) solution (pH 8.5). The CH_2_Cl_2_ fraction was dried using anhydrous Na_2_SO_4_, filtered, and concentrated under reduced pressure. The solid residue was purified by flash column chromatography (silica gel 230–400 mesh) with an EtOEt: EtOAc gradient (80:20 to 60:40) to obtain *2-methoxy-4-(5-(thiophen-2-yl)-1,3,4-oxadiazol-2-yl)phenol* (A_1_B_4c_, 2 mg, 23.3%). *Rf* 0.30 (CH_2_Cl_2_: MeOH = 9.85:0.15). ^1^H-NMR (CDCl_3_, 300 MHz), *δ* (ppm) 4.02 (s, 3H_a_), 6.02 (s, 1H_b_), 7.04 (d, *J* = 8.1 Hz, 1H_c_), 7.19 (dd, *J* = 5.0, 3.8 Hz, 1H_d_), 7.56 (dd, *J* = 5.0, 1.2 Hz, 1H_e_), 7.62 (m, 1H_f_), 7.66 (m, 1H_g_), 7.82 (dd, *J* = 3.6, 1.2 Hz, 1H_h_). ^13^C-NMR (CDCl_3_, 75 MHz), δ (ppm) 56.3 (C_1_), 109.2 (C_2_), 114.9 (C_3_), 115.9 (C_4_), 121.1 (C_5_), 125.4 (C_6_), 128.1 (C_7_), 129.6 (C_8_), 129.9 (C_9_), 146.9 (C_10_), 149.1 (C_11_), 160.5 (C_12_), 164.1 (C_13_). HRMS *m*/*z* (ES+) found 275.0501 (100%), C_13_H_10_N_2_O_3_S requires [M + H]^+^ 275,0490. m.p. 224°C. Analytical HPLC-UV (Supplementary Figure S8), rt. 2.16 min, purity >98%.

*(E/Z)-N′-((5-(hydroxymethyl) furan-2-yl) methylene) thiophene-2-carbohydrazide* (A_5_B_4_): An EtOH solution (2 mL) of thiophene-2-carboxylic hydrazide (142.2 mg, 1 mmol, 1 eq.) was mixed with an EtOH solution (2 mL) of 5-(hydroxymethyl) furan-2-carbaldehyde (126.1 mg, 1 mmol, 1 eq.) and 2 μL of TFA was added. The resulting solution was mixed for 18 h at room temperature. 4 mL of distilled water was added, and white crystals were produced and collected by filtration. The white crystals were washed with cold EtOH and dried under reduced pressure. A_5_B_4_ yield 70%. *Rf* 0.27 (CH_2_Cl_2_: MeOH = 9.5:0.5). ^1^H-NMR (DMSO-_d6_, 300 MHz), *δ* (ppm) 4.46 (s, 2H_a_), 5.40 (br s, 1H_b_), 6.45 (d, *J* = 3 Hz, 1H_c_), 6.87 (d, *J* = 3 Hz, 1H_d_), 7.22 (t, *J* = 4.3 Hz, 1H_e_), 7.87–7.95 (m, 2H_f-g_), 8.08 (br s, 1H_h_), 8.26 (b s, 1H_h_ isomer), 11.77 (s, 1H_i_). ^13^C-NMR (DMSO-_d6_, 75 MHz), δ (ppm) 55.7, 109.2, 114.9, 128.0, 128.9, 131.9, 134.8, 137.1, 138.2, 148.5, and 157.9. HRMS *m*/*z* (ES+) found 251.0491 (100%), C_11_H_10_N_2_O_3_S requires [M + H] + 251.0490. m.p. 212°C.

*(5-(5-(thiophene-2-yl)-1,3,4-oxadiazol-2-yl)furan-2-yl)methanol* (A_5_B_4c_): (E/Z)-N′-((5-(hydroxymethyl) furan-2-yl) methylene) thiophene-2-carbohydrazide (27 mg, 0.11 mmol) and (diacetoxyiodo)benzene (DIB) (59 mg, 0.18 mmol) in CH_2_Cl_2_: MeOH (20 mL: 1.3 mL) at room temperature. After 21 h of reaction, the solvents were evaporated, and the residue was washed with EtOAc. The residue was then dried under reduced pressure and dissolved in cool EtOH (10 mL) for crystallization. A white solid was obtained and purified by column chromatography (silica gel 230–400 mesh) with hexane (Hex)-EtOAc in a gradient (from 80:20 to 60:40) to obtain A_5_B_4c_. Yield, 52%. *Rf* 0.20 (Hex-EtOAc = 6.0:4.0). ^1^H-NMR (CDCl_3_, 300 MHz), δ (ppm) 4.75 (s, 2H_a_), 6.52 (d, *J* = 3.6 Hz, 1H_b_), 7.17 (d, *J* = 3.6 Hz, 1H_c_), 7.19 (dd, *J* = 5.1, 3.9 Hz, 1H_d_), 7.58 (dd, *J* = 5.1, 1.2 Hz, 1H_e_), 7.83 (dd, *J* = 3.9, 1.2 Hz, 1H_f_). ^13^C-NMR (CDCl_3_, 75 MHz), δ (ppm) 57.28 (C_1_), 109.91 (C_2_), 115.18 (C_3_), 128.26 (C_4_), 130.53 (C_5_), 130.19 (C_6_), 124.60 (C_7_), 138.62 (C_8_), 156.80 (C_9_), 158.20 (C_10_), and 160.50 (C_11_). HRMS *m*/*z* (ES+) found 249.0336 (100%), C_11_H_8_N_2_O_3_S requires [M + H] + 249.0334. m.p. 220°C. Analytical HPLC-UV (Supplementary Figure S9), rt. 1.17 min, purity >97%.

All NMR spectra and chromatograms of the synthesized compounds are shown in Supplementary Figure S10.

### 2.2 Recombinant protein purification

As previously reported, the versions of *Tc*BDF3 were purified from inclusion bodies (Alonso et al., 2016). Briefly, the plasmids pDEST17-*Tc*BDF3, pDEST17-*Tc*BDF3m, and pDEST17-*Tc*BD3 were transformed into *Escherichia coli* BL21, and the recombinant proteins (fused to a His tag) were obtained by induction with 0.1-mM isopropyl-*β*-d-thiogalacto-pyranoside (IPTG) overnight at 22°C. Protein was purified from inclusion bodies and solubilized proteins were dialyzed against 0.1-mM phosphate buffer pH 8. *Tc*BD2 was purified under soluble conditions, as reported previously from *E. coli* BL21 transformed with pDEST17-*Tc*BD2. The recombinant protein was obtained by induction with 0.5-mM IPTG for 3 h at 37°C (Tavernelli et al., 2024). The recombinant protein was purified using a nickel-charged resin (Ni-NTA Agarose, Qiagen) following the manufacturer’s instructions. Correct folding was verified by circular dichroism spectroscopy using a spectropolarimeter (Jasco J-810, Easton, MD, USA).

### 2.3 Tryptophan intrinsic fluorescence quenching assay

96-well black microplates with 10-μM recombinant proteins (*Tc*BDF3, *Tc*BDF3m, *Tc*BD3, and *Tc*BD2) and three concentrations of each compound were used (5, 10, and 20 μM). In this assay, A1B4 and iBET-151 (positive control) were used. All determinations were made from the top with an excitation wavelength of 275 and a 340/30 nm emission filter, 50 reads *per* well, and a PMT-sensitivity setting of 180 in a Synergy HT multidetection microplate reader equipped with time-resolved capable optics. Each condition was tested in triplicates, and normalized maximum fluorescence intensityvs. Concentration was plotted using GraphPad Prism 9.0. The relative fluorescence intensity obtained to the condition without the inhibitor was normalized. Compounds that showed concentration-dependent fluorescence quenching, selecting a cut-off value of 0.9 normalized units, were considered positive binders.

### 2.4 Thermal shift assay

The proteins were buffered in 10-mM HEPES, pH 7.5, and 500 mM NaCl and assayed in a 48-well plate at a final concentration of 2 μM in a volume of 20 μL. Compounds were added at a final concentration between 1 and 200 μM to calculate dissociation constants. In this assay, A1B4, A1B4c, A5B4 and A5B4c were used. SYPRO Orange (Invitrogen) was added as a fluorescence probe at a dilution of 1:1000. The excitation and emission filters for the SYPRO Orange dye were set at 465 and 590 nm, respectively. The temperature was increased with steps of 2°C/min from 25°C to 96°C, and fluorescence readings were taken at each interval in a CFX Opus 96 Real-Time System (Biorad) and analyzed using Maestro Software (Biorad). The melting curves and average melting temperature for each condition were obtained and plotted in temperature vs. concentration graphs in GraphPad Prism 9.0. Plots were fitted using the differential fluorescence scanning (DSF) single binding site equation to obtain the dissociation constants (*K*_d_) in GraphPad Prism 9.0.

### 2.5 Fluorescence polarization (FP) assay

FP measurement was performed on a Cary Eclipse spectrophotometer equipped with a polarizer at an excitation wavelength of 488 nm and an emission wavelength of 675 nm in a 3-ml fluorescence cuvette. The essay was set at a final volume of 2 mL, 0.5 μM of BSP-AF488, 100-μM *Tc*BDF3, phosphate pH = 8, glycerol 1%, and DMSO 0.5%. The FP values were plotted against the log of the compound concentrations in a non-linear regression model in GraphPad Prism 9.0 as previously described (Zhang et al., 2002). In this assay, A1B4, A1B4c, and A5B4c were used. Briefly, free and bound probes (BSP-AF488 in the presence of *Tc*BDF3) were included in each replica. Since the intensity of the probe remains similar for all samples, the fraction of the probe bound to *Tc*BDF3 is correlated to the mP value. Thus, the percentage of inhibition can be derived from the equation % inhibition =100[1 − (mP − mPf)/(mPb − mPf)], in which mPf is the free probe control and mPb is the bound probe control. Based on the percentage of inhibition, the inhibitory concentration (IC_50_), or the inhibitor concentration at which 50% of the bound probe is displaced, is obtained by fitting the inhibition data.

### 2.6 Beta-galactosidase assay

*T. cruzi* Dm28c that expresses the *E. coli* LacZ gene epimastigotes, trypomastigotes, and amastigotes were cultured and obtained as previously described (Buckner et al., 1996; Alonso et al., 2021). The compounds were dissolved in DMSO with a final concentration of the solvent below 1% to ensure that DMSO does not interfere with cytotoxicity. The controls included for the amastigotes (intracellular stage) were (a) uninfected cells and (b) infected with 1 μg/mL benznidazole (BZ) (positive control) and 1% DMSO to evaluate the toxicity of the solvent. For epimastigotes and trypomastigotes, the controls used were: 1-μg/ml benznidazole (BZ) (positive control) and 1% DMSO to evaluate solvent toxicity. Results obtained with this assay were verified by manually counting the parasites. The results were expressed as the percentage of inhibition of *T. cruzi* growth in compound-treated infected cells compared to untreated infected cells for amastigotes. For epimastigotes and trypomastigotes, treated and untreated cultured parasites were compared. Quadruplicates were run in the same microplate and repeated in three experiments. Half-maximal IC_50_ values were calculated using non-linear regression on GraphPad Prism 9.0, as previously described (Alonso et al., 2021).

### 2.7 MTT assay

The 3-(4,5-dimethylthiazol-2-yl)-2,5-diphenyltetrazolium bromide (MTT) reduction assay determined cell viability after treatment as previously described (García et al., 2018). Optical density (OD) was spectrophotometrically quantified (*λ* = 540 nm) using a Synergy HT multidetection microplate reader. DMSO was used as blank, and each treatment was performed in triplicates. The 50% cytotoxic concentration (CC_50_) values were calculated by non-linear regression on GraphPad Prism 9.0, and the selectivity indexes (SI) were determined as the ratio CC_50_/IC_50_ in all three life cycle stages.

### 2.8 Structural modeling and docking

The initial model of *Tc*BDF3 was generated with AlphaFold2 using ColabFold with the following options: --num-models 5 --num-recycle 10 --rank plddt --num-relax 1 --use-gpu-relax, and the sequence of *Tc*BDF3 from Dm28c (Jumper et al., 2021). A custom multiple sequence alignment with the combination of the alignments produced with the Discoba database and the default alignment of the ColabFold server was used, as described by Wheeler (2021). All models obtained showed high average pLDDT values (93.6, 93.1, 92.7, 92.6, and 92.1) and pTM (0.840, 0.829, 0.828, 0.827, and 0.827). Domain cavity sizes were computed with CAVIAR (Marchand et al., 2021). The structural alignment of the ET domain of *Tc*BDF3 and other ET domains was performed using the ChimeraX matchmaker algorithm guided by Smith–Waterman alignments (Meng et al., 2023).

Docking was done using Glide in extra precision (XP) mode. *Tc*BDF3 structure was obtained from a representative molecular dynamics (MD) simulation snapshot. Ligand docking used Schrödinger software (version 2023-3). Protein preparation employed the Protein Preparation Wizard within Schrödinger software. LigPrep was used to prepare ligands. The grid box of 20 Å was centered in the binding site. The best docking pose of each ligand was selected for further analysis.

### 2.9 Molecular dynamics simulations

Molecular dynamics (MD) simulations were performed using Amber22 software (Case et al., 2023). The starting structure of the protein was obtained using AlphaFold2 (see above), and the structures of the protein with the ligands were taken from the docking studies. Protein and ligand-protein complexes were subjected to the 100 ns MD simulation protocol using the particle mesh Ewald molecular dynamics (PMEMD) CUDA software implemented in Amber22 with the FF14SB force field. Ligand parameters (van der Waals radius, force constants, etc.) were from the gaff database. Partial charges were RESP charges computed using the Hartree–Fock method and 6-31G* basis set (Bayly et al., 1993). The proteins were first immersed in a periodical octahedral box of TIP3P water molecules; structures were then optimized, taken from 0 to 300 K at constant volume for 100 ps, equilibrated at a constant pressure of 1 bar for 200 ps, and finally subjected to 100 ns of production simulation. Temperature control was performed using the Langevin thermostat, and pressure control was performed using the Monte Carlo barostat (Gordon et al., 2009). The binding-free energy of each complex and its decomposition per residue were performed with the Poisson-Boltzmann Surface Area of Molecular Mechanics (MM-PBSA) in AmberTools (Faller and de Pablo, 2001; Wang et al., 2016; Case et al., 2023).

### 2.10 Phylogenetic analysis

The structural homologous sequences of *Tc*BDF3 were retrieved by combining structural and profile HMM searches with strict cutoffs. First, a PDB file was generated with the extraterminal (ET) domain from *Tc*BDF3 AF2 model, and its atom coordinates were used as a query on the Foldseek search server (van Kempen et al., 2023) against AlphaFold/UniProt50 v4 database (50% sequence identity clusters). This generated a list of all non-redundant proteins that contained an ET fold in its AF2 structure. Only proteins with a probability greater than 0.9 were considered. The HMMER v3.4 search algorithm (Eddy, 2011) was used to detect the Pfam bromodomain (BD) profile HMM PF00439 in the sequences, extracting their starting and end positions and the number of matches (i.e., the number of bromodomains), considering only those with a score bigger than 30; resulting in a list of 307 proteins with at least one BD before ET. No BD was detected at the C-terminal of the ET in any sequence. Then, the last BD of each sequence was concatenated with its corresponding ET, followed by a sequence redundancy filter of 90%, leaving 121 proteins. Each domain was then aligned separately with ClustalO (Sievers and Higgins, 2018) using default settings, followed by removing proteins without complete coverage of the BD sequence and a per-domain realignment. Non-informative positions were removed with ClipKIT (Steenwyk et al., 2020) using the kpic-gappy method. Finally, a maximum likelihood tree was generated using IQ-TREE2 (Minh et al., 2020), with a bootstrap of 1,000, letting ModelFinder find the best substitution model. Tree visualizations were performed with a Dendroscope (Huson and Scornavacca, 2012).

## 3 Results and discussion

### 3.1 A1B4 binds to different versions of *Tc*BDF3

**Figure 1:**
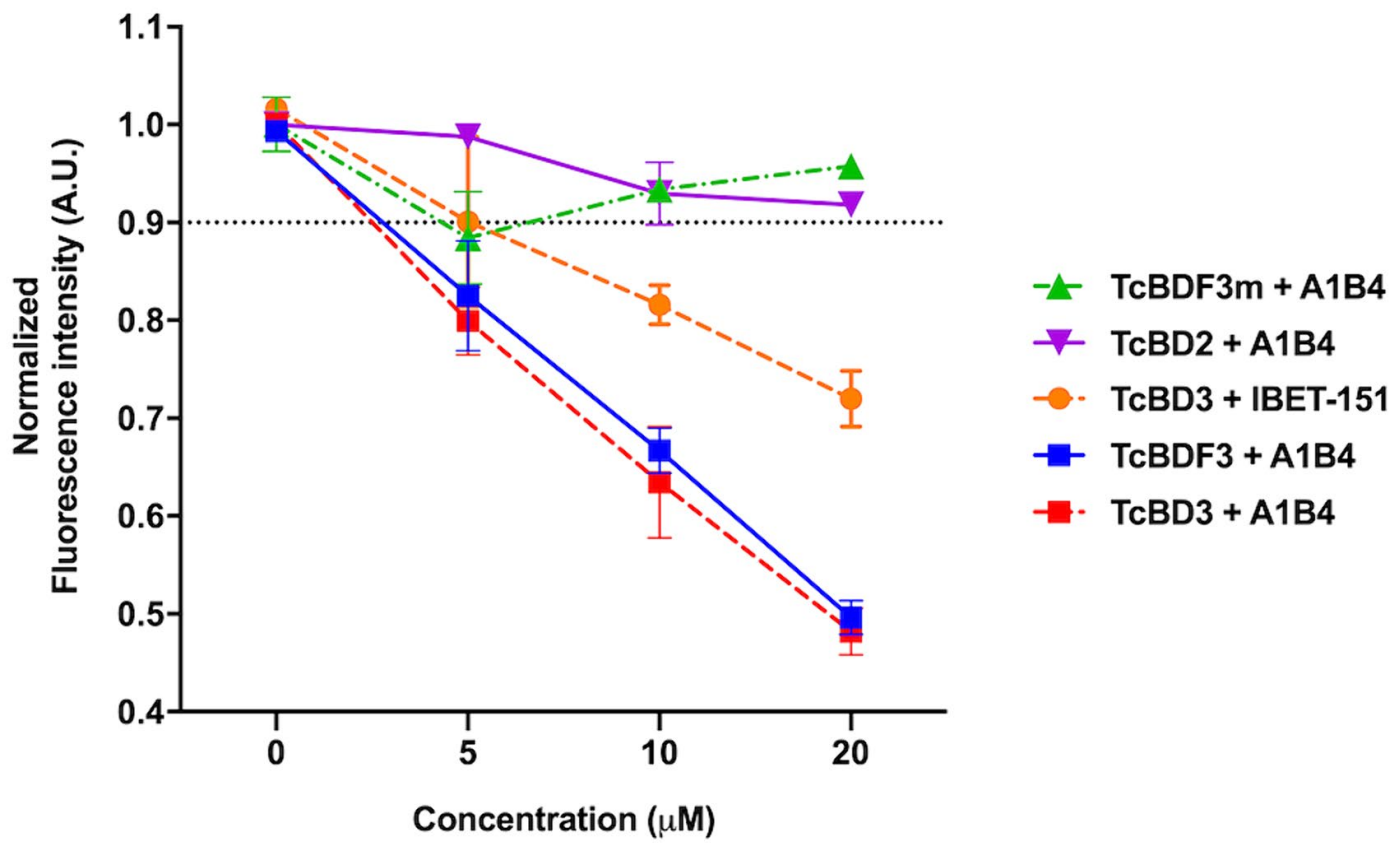
In-house fluorescence quenching assay using the recombinant full-length protein (*Tc*BDF3), only the bromodomain (TcBD3), the mutated full-length version (*Tc*BDF3m), and the bromodomain from *Tc*BDF2 (*Tc*BD2). We measured the intrinsic fluorescence of each bromodomain construction with three increasing concentrations of A1B4 and, as a positive control, the BET-bromodomain inhibitor iBET-151 (previously reported to bind to *Tc*BDF3). The relative fluorescence at each concentration was measured in triplicates, and the mean and standard deviation were plotted. The black dashed line indicated the normalized fluorescence intensity cut-off value used to determine positive binding.

As mentioned in the Introduction, our group previously described A1B4, a compound that binds to *Tc*BDF3 *in vitro* and displays trypanocidal activity (García et al., 2018). Later, Laurin et al. (2021) reported contradictory results regarding the binding of A1B4 to a recombinant bromodomain portion of *Tc*BDF3 (*Tc*BD3) using waterLOGSY. A possible explanation for the reported difference between Laurin et al.’s results and ours is that they used a different recombinant bromodomain. In contrast, the full-length protein (*Tc*BDF3) was used in our publication. To further evaluate the significance of this difference, a truncated version of *Tc*BDF3 containing only the bromodomain (*Tc*BD3: amino acids 1–151) was used, together with the full-length version to evaluate A1B4 binding using our in-house fluorescence quenching assay (Figure 1). As an additional control experiment, the binding of A1B4 to a mutated version of *Tc*BDF3 (*Tc*BDF3m) that includes two-point mutations (Y123A and L130A) was measured. *Tc*BDF3m was designed to retain its secondary structure while losing its ability to bind to its acetylated ligand *in vitro* (Alonso et al., 2016). Furthermore, A1B4 binding to the recombinant bromodomain of *Tc*BDF2 (*Tc*BD2) was measured to explore selectivity. *Tc*BDF2 is a nuclear bromodomain-containing protein that recognizes acetylated histone H4, H2B, and H2B.V in *T. cruzi* (Villanova et al., 2009; Pezza et al., 2022). A1B4 did not bind to *Tc*BDF3m or *Tc*BD2, indicating high selectivity for the bromodomain portion of *Tc*BDF3 (*Tc*BD3) (Figure 1). As a positive binding control, iBET-151 was used, a BET bromodomain inhibitor previously reported to bind to *Tc*BDF3 (Alonso et al., 2016). This assay validated our previous results and confirmed that A1B4 binds to the bromodomain of *Tc*BDF3 with specificity.

**Figure 2:**
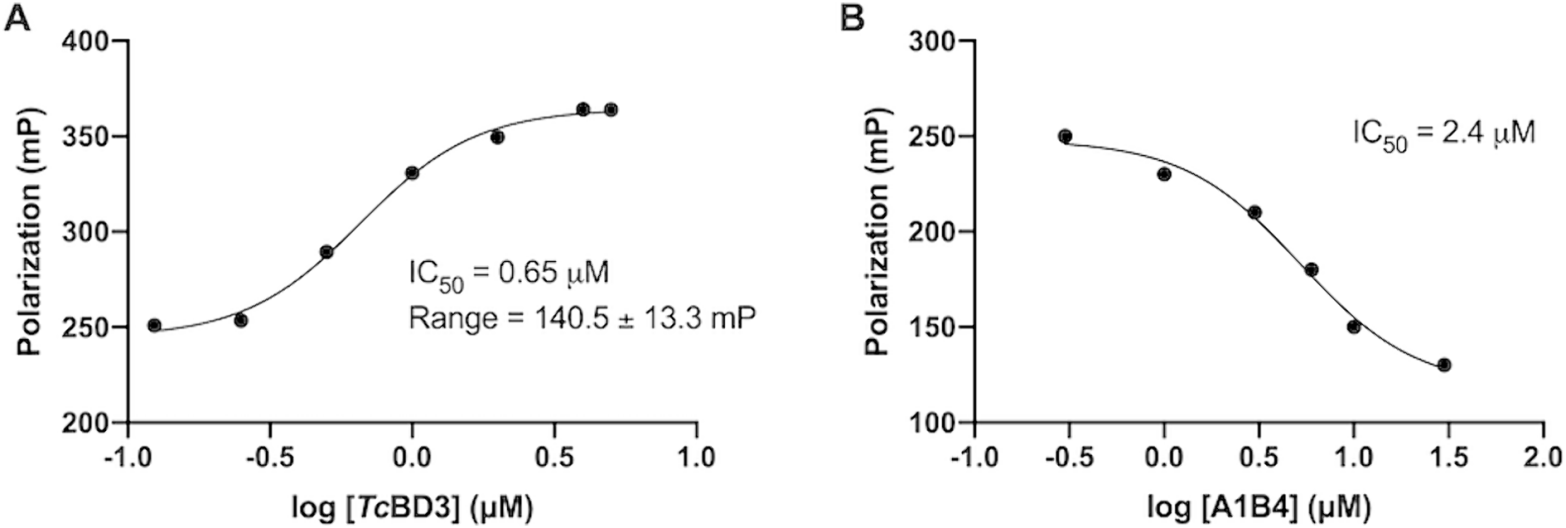
**(A)** Binding of *Tc*BDF3 to BSP-Alexa 488. An increasing amount of *Tc*BDF3 protein was added to 1 mM of BSP-Alexa 488. Fluorescence polarization was measured at room temperature. Data was analyzed in GraphPad Prism 9.0 by fitting mP data using a sigmoidal dose–response non-linear regression curve. **(B)** A1B4 was titrated against a constant concentration of BSP-Alexa-488 (0.5 mM) in the presence of 100-μM *Tc*BDF3. Data was analyzed in GraphPad Prism 9.0 by fitting mP data using a sigmoidal dose–response non-linear regression curve.

In our previous study, the dissociation constant (*K*_d_) reported for A1B4 and *Tc*BDF3 was 1.7 μM calculated using DSF, also known as thermal shift (Table 1). To further validate the parameters obtained, a Fluorescence Polarization (FP) assay recently described by our group for *Tc*BDF2 was used (Tavernelli et al., 2024). FP is a sensitive technique that allows quantitative analysis of the interaction between two molecules. This assay employs a fluorescent probe (AlexaFluor-488) linked to bromosporine (BSP-AF488). Bromosporine is a mammalian bromodomain pan inhibitor, which has been reported to bind to *Tc*BDF3 and *Tc*BDF2 (Alonso et al., 2016). First, the dissociation constant of BSP-AF488 and *Tc*BDF3 was determined and a working window of approximately 140 mP was obtained, which was necessary to proceed with the binding assay (Figure 2A). The FP was then measured using a fixed concentration of *Tc*BDF3 and increasing amounts of A1B4 to obtain a binding activity of 2.4 μM for A1B4, in agreement with the value previously calculated by DSF (Table 1) (Figure 2B). These results confirmed the binding of A1B4 to *Tc*BDF3 and validated this small molecule as a starting point for the search for new 1,3,4-oxadiazoles as parasitic bromodomain inhibitors. Second, a structural analysis of *Tc*BDF3 and *Tc*BD3 was performed *in silico* to explain the differences reported in the literature.

**Table 1:** Binding parameters for *Tc*BDF3 by thermal shift and fluorescence polarization*.

Compound	Structure	*K*_d_ ± SD DSF (μM)	IC_50_ ± SD FP
A1B4	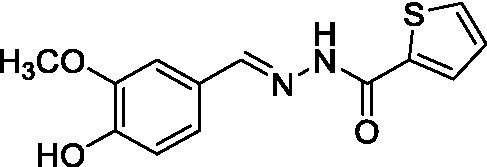	1.7 ± 0.2 (García et al., 2018)	2.4 ± 0.3
A5B4	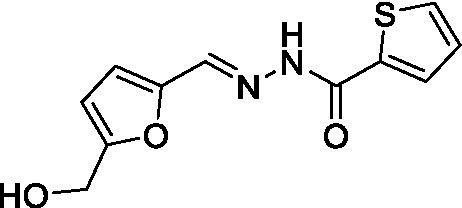	136 ± 2.4	n.d.
A1B4c	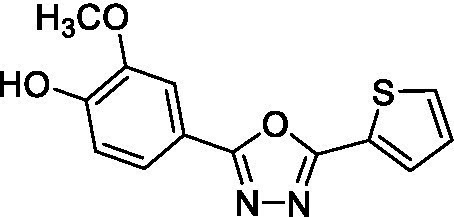	4 ± 0.3	8.4 ± 0.5
A5B4c	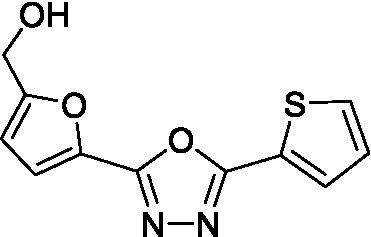	4.8 ± 0.2	10.5 ± 0.9

^*^K_d_ for A1B4 by DSF corresponds to that already published (García et al., 2018). The results are expressed with the SD obtained from three independent experiments. Each experiment was performed in triplicates. CC_50_, cytotoxic concentration; DSF, differential fluorescence scanning; FP, fluorescence polarization; n.d., not determined; SD, standard deviation.

### 3.2 The binding cavity of *Tc*BDF3 changes its volume and hydrophobicity in the truncated version

**Figure 3:**
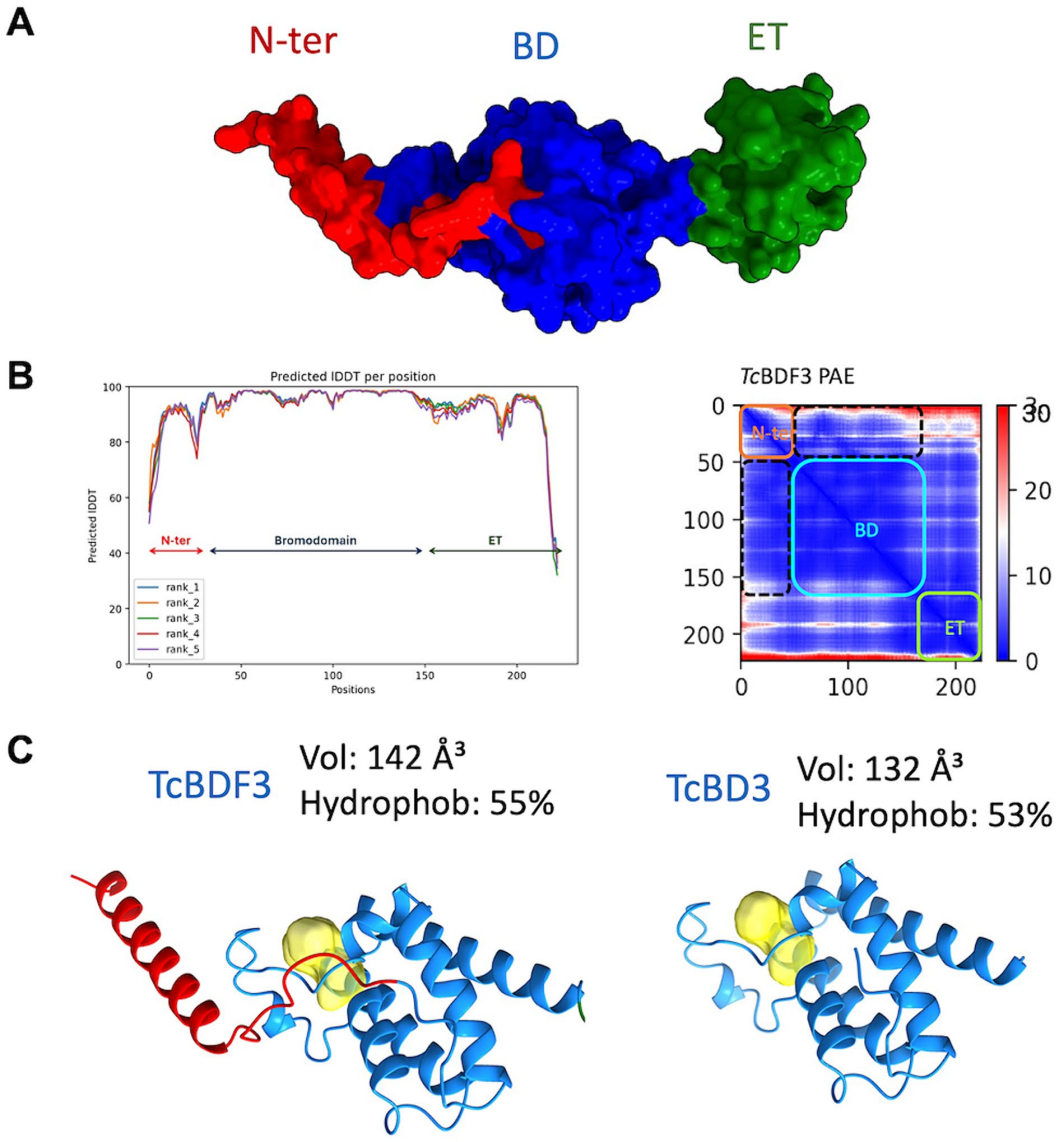
**(A)** Structure of *Tc*BDF3 predicted by AlphaFold2 (surface representation). N-ter, N-terminal *α*-helix (red); BD, Bromodomain (blue); ET, extraterminal domain (green). **(B)** Predicted IDDT per position for the five models obtained and Predicted Aligned Error (PAE) plot obtained from AF2 prediction of *Tc*BDF3 structure. Dash lines indicate the interface between the BD and the N-tern α-helix in the PAE plot. In the plot blue color indicates lower PAE, which means the best prediction of relative ubication of the amino acids. **(C)** Volume and hydrophobicity of BD cavity from full-length *Tc*BDF3 and isolated bromodomain (*Tc*BD3) calculated with CAVIAR.

To investigate the discrepancies in the binding parameters reported for the full-length and truncated versions of *Tc*BDF3, we generated a reliable structural model of *Tc*BDF3 using AlphaFold2, enhanced by the Discoba database. This database was specifically developed to improve the quality of predictions on protein structure within the phylogenetic group that includes trypanosomatids (Figure 3A; Wheeler, 2021). To evaluate the quality of the predicted structure, the pLDDT values per residue were plotted, and high values across the entire protein length for all the models were observed, both over the BD region and the ET region (Figure 3B, left panel). In addition, the high pTM and low domain error values in the PAE matrix graphed in Figure 3B (right panel) indicated a compact globular structure. When the 3D structure for the full-length and the truncated protein was simulated using AlphaFold2 it was observed that the bromodomain portion of *Tc*BDF3 has a slightly less hydrophobic pocket with a more extensive cavity (142 Å^3^) compared to the pocket of the isolated domain (132 Å^3^) (Figure 3C). This could be explained by a conserved *α*-helix formed by the first 32 amino acids of the *N*-terminal portion that interact tightly with the BD or ZA loops, which can impact its mobility. The fact that the inner structure is not precisely conserved when the bromodomain is expressed in isolation could explain, in part, the discrepancies in binding observed in the literature (Laurin et al., 2021). It is worth mentioning that, although in the same order of magnitude, the observed binding parameters for A1B4 for the truncated version are slightly higher than that of the complete protein (Supplementary Figure S1).

### 3.3 1,3,4-oxadiazoles derived from A1B4 bind to *Tc*BDF3 *in vitro*

With these results in hand, the validated methodology was applied to three other compounds related to A1B4: hydrazone A5B4 and 1,3,4-oxadiazoles A1B4c and A5B4c. Hydrazone A5B4 was included as a control because it contains the thiophene portion observed in A1B4 and a furane ring observed in another *Tc*BDF3 binder previously discovered by our group (Ramallo et al., 2018). Although A5B4 was present in the dynamic library that led to the discovery of A1B4, it was not amplified in the presence of *Tc*BDF3 (García et al., 2018). The other 1,3,4-oxadiazoles are more rigid versions of their hydrazone precursors, A1B4 and A5B4, respectively. Furthermore, 1,3,4-oxadiazoles represent an engaging heterocyclic platform found in products that show a wide range of biological properties (Gao and Wei, 2013; Chauhan et al., 2023).

Hydrazone A5B4 was prepared from the corresponding acyl hydrazide and aldehyde in the presence of TFA. 1,3,4-oxadiazoles A1B4c and A5B4c were synthesized from the corresponding hydrazones, by oxidative cyclization with chloramine T or (diacetoxyiodo)benzene, respectively (see Methods section). A comparison of the binding parameters for the hydrazones A1B4 and A5B4 (Table 1) enables us to explain why the latter compound was not amplified in our previous study while A1B4 was. The affinity of A5B4 affinity for *Tc*BDF3 (*K*_d_ = 136 μM) is significantly lower than that of A1B4 (*K*_d_ = 1.7 μM). The cyclic compounds gave slightly higher dissociation constants but in the same order of magnitude as A1B4 using both DSF and FP (Table 1; Supplementary Figure S2). It should be noted that cyclization of A5B4 led to a significantly improved affinity for *Tc*BDF3 (Table 1). The results were of the same order of magnitude for the full-length and truncated version of *Tc*BDF3 (Supplementary Figure S1).

### 3.4 1,3,4-oxadiazoles have an improved trypanocidal effect

To evaluate the *in vivo* effect of the new compounds, the cytotoxic effect against the three life cycle stages of *T. cruzi* (epimastigotes, trypomastigotes, and amastigotes) *in vitro*, and a mammalian cell line was determined for all of the small molecules. For these *in vivo* cell assays, a *T. cruzi* Dm28c strain expressing the *E. coli β*-galactosidase as a reporter gene was used (Alonso et al., 2021). Epimastigotes, trypomastigotes, or amastigotes obtained 48 h postinfection of Vero cells monolayers, were incubated with the compounds. As shown in Table 2, A1B4c has a cytotoxic effect of <10 μM in all life cycle stages, improving the parasitic activity of A1B4. A5B4c was more active than A1B4 but to a lesser extent than A1B4c. Depending on the stage, the selectivity indexes (SI) obtained ranged between 11 and 54 for A1B4c, and between 21 and 37 for A5B4c, thereby improving upon those obtained for A1B4 (Table 2).

**Table 2:** IC_50_ in all life cycle stages and selectivity indexes (SI)*.

IC_50_ ± SD (μM)	Epimastigotes	Tripomastigotes	Amastigotes	Vero cells (CC_50_)	SI (E/T/A)
A1B4 (García et al., 2018)	23 ± 2.04	17.8 ± 2.13	13.1 ± 1.43	269 ± 4.28	11.7/15/20.5
A1B4c	8.13 ± 0.87	1.67 ± 0.34	6.9 ± 0.67	90.8 ± 3.54	11/54.4/13
A5B4c	14.8 ± 1.38	13.42 ± 1.25	8.49 ± 1.63	315.6 ± 4.35	21.3/23.5/37

*IC_50_ was calculated with a Dm28c strain expressing *β*-galactosidase as indicated in the Methods section. CC_50_ for Vero cells was determined using MTT. The values presented for A1B4 compounds are those already published. The results are expressed with the SD obtained from three independent experiments. Each experiment was performed in triplicates. A, amastigotes; CC_50_, cytotoxic concentration; E, epimastigotes; T, trypomastigotes; SD, standard deviation.

### 3.5 1,3,4-oxadiazoles bind to the ac-lysine binding pocket of *Tc*BDF3

**Figure 4:**
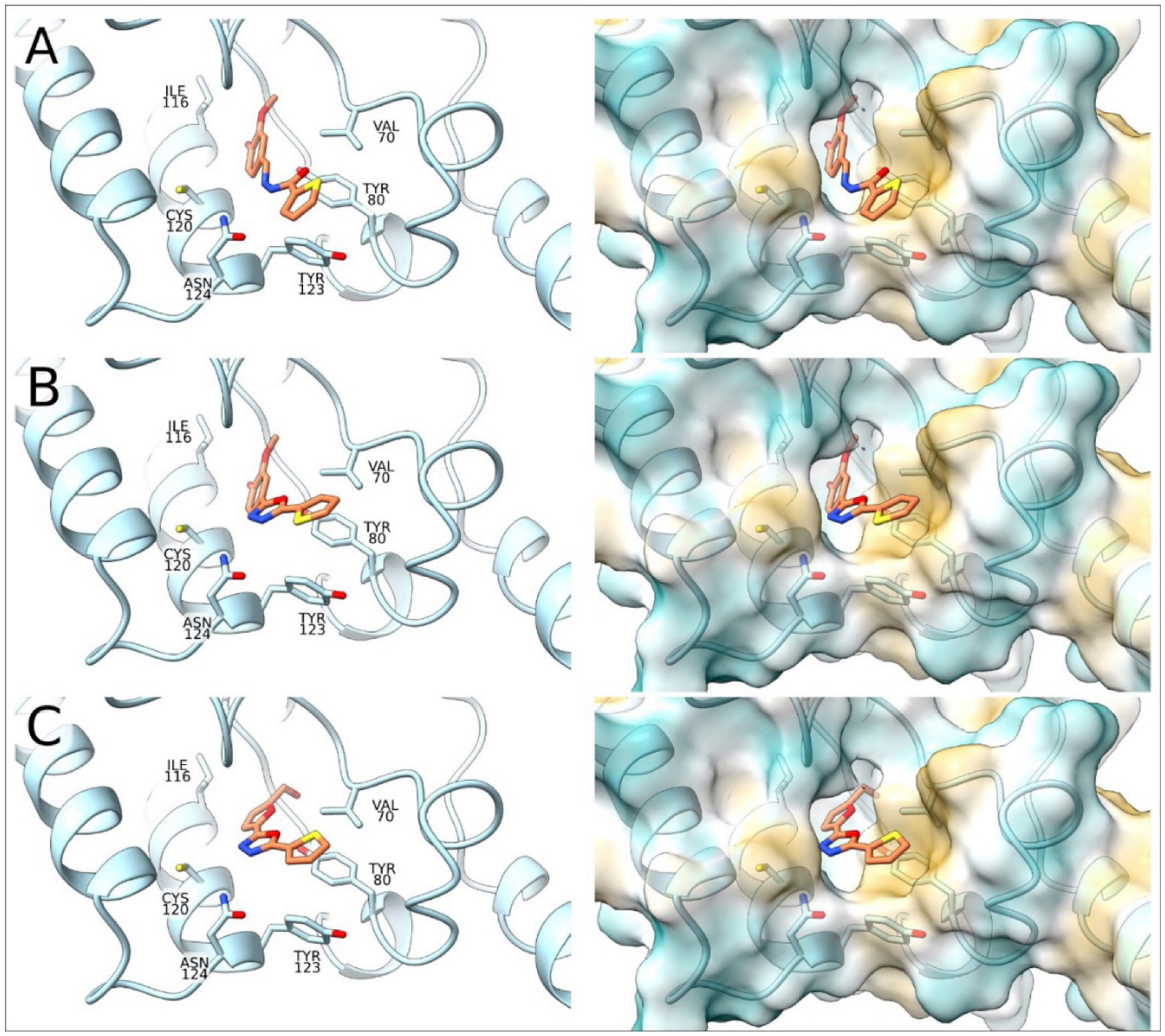
Molecular docking of *Tc*BDF3-ligand structures obtained from molecular dynamics simulations. In the left panel, the C atoms of the ligands are shown in orange and the C atoms of *Tc*BDF3 are in light blue. O atoms are colored in red, N atoms in blue, and S atoms in yellow. In the right panel, *Tc*BDF3 is represented as a surface and the ligands are depicted in liquorice format. **(A)** A1B4, **(B)** A1B4c, and **(C)** A5B4c.

To complement the activity studies, molecular docking was employed to understand the binding of A1B4, A1B4c, and A5B4c to the bromodomain at the atomistic level. Figure 4 shows the docking results obtained, which determine that the three ligands are located inside the hydrophobic pocket of the bromodomain (responsible for the Ac-Lysine binding) interacting with Val70, Leu75, Tyr80, Ile116, Asn119, Cys120, Tyr123, and Asn124 of *Tc*BDF3. The ligands are detected with the thiophene group toward the outside of the protein. The position of the thiophene group is vital in achieving sulfur-*π* interactions with Tyr80, and this type of interaction is crucial for the binding processes. The alcohol group of the ligands is placed toward the inside of *Tc*BDF3 and allows interaction with Asn119, Tyr123, and Asn124 (Figure 4).

Furthermore, the *Tc*BDF3-ligand structures obtained from the docking studies were subjected to molecular dynamics (MD) simulations to evaluate the conformation of the complexes as a function of time. Results show that the three ligands remained in the cavity and maintained the interactions described above (Supplementary Figure S3). In addition to the temporal evolution of the complexes, the MD simulations can provide thermodynamic data, so the binding energies for the three complexes were calculated. A more favorable binding for A1B4 compared to A1B4c and A5B4c (ΔG [A1B4/A1B4c] = 4.52 kcal/mol, ΔG [A1B4/A5B4c] = of 5.97 kcal/mol) was estimated. These results correlate with the dissociation constants obtained by DSF and FP. The binding energy was further decomposed into the contribution of each residue, and the results are presented in Supplementary Figure S4, where only the residues of the binding site were considered. In all three cases, the contribution to the binding energy follows the same pattern and is consistent with the binding mode analyzed by the docking studies.

### 3.6 *Tc*BDF3 is an atypical member of the BET family

According to Laurin et al., one possible explanation for the lack of binding observed for A1B4 to a truncated version of *Tc*BD3 that does not contain the *N*-terminal *α*-helix is that A1B4 could be binding to a second site of *Tc*BDF3, distinct from the hydrophobic pocket of the BD. To test this hypothesis, we explored the *Tc*BDF3 modeled structure in search of additional binding sites using CAVIAR, finding one additional smaller pocket in the C-terminal half of the protein (Supplementary Figure S5). The size of this cavity (101 Å^3^) and the fact that A1B4 and the two cyclic derivatives displaced the bromodomain-specific bromosporine probe in the FP assay using both full-length and our C-terminal truncated version make us dismiss the possibility that they are interacting with this alternative domain. Moreover, docking simulations do not predict the insertion of any of the compounds into the cavity of this alternative domain. Given that our recombinant *Tc*BD3 version contains the conserved *N*-terminal α-helix, the discrepancy in binding affinity observed by Laurin et al. could be attributed to the absence of this structural element in their truncated protein. This *N*-terminal α-helix likely contributes to stabilizing the bromodomain structure, mainly through stabilization of the ZA loop, influencing the binding pocket’s conformation and, consequently, the affinity of a small molecule ligand such as A1B4. Since most BD research centers on the development of inhibitors using isolated BDs, it is possible that removing specific regions surrounding the domain could affect the binding affinity. Therefore, it might be advantageous to keep specific non-BD areas, especially those that interact with the ZA or BC loops, as these elements may play a crucial role in stabilizing the BD structure and influencing the ligand binding.

On closer inspection, it was observed that the C-terminal region has an extraterminal (ET) domain fold, found in the BET (bromodomain and extraterminal Domain) family of bromodomains (Taniguchi, 2016). The structural alignment between the ET domain of *Tc*BDF3 and the ET domain of human BRD2 previously characterized is shown in Supplementary Figure S5 with their HHpred values. In addition, the *Tc*BDF1 structure was predicted to have an ET fold (data not shown). These observations suggest that *Tc*BDF1 and *Tc*BDF3 could be part of this family.

**Figure 5:**
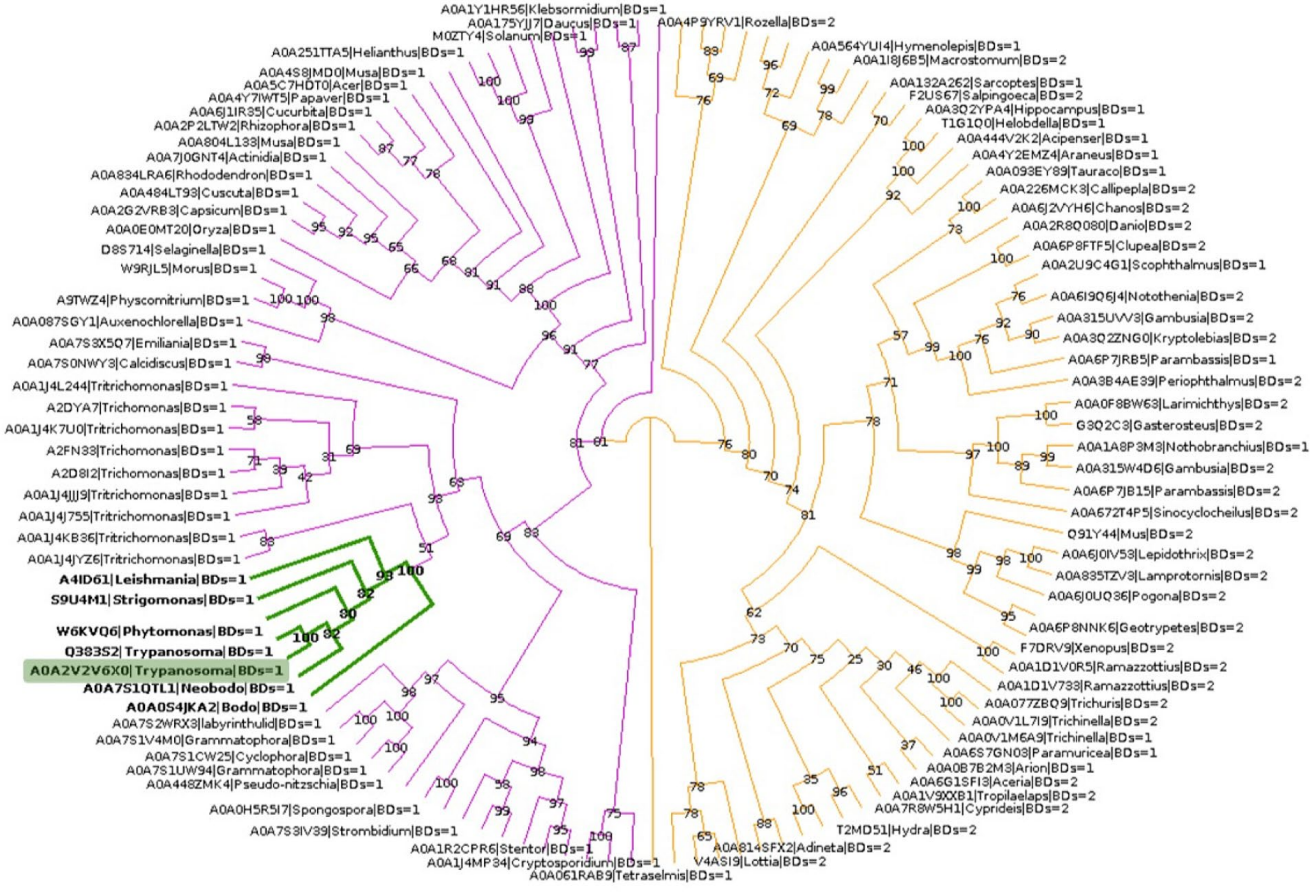
BET proteins phylogenetic analysis. *Tc*BDF3 is highlighted in green, and green branches correspond to kinetoplastids. Purple branches contain proteins with only one BD, which includes kinetoplastids sequences. Orange branches contain clades of BET proteins with either one or two BDs.

To explore the phylogenetic relationship between *Tc*BDF3 and other BET proteins, homologous sequences of *Tc*BDF3 were recovered by combining structural and profile HMM searches with strict cut-off values (Supplementary Figure S6A). The final sequence dataset consisted of 121 non-redundant proteins from the four eukaryotic kingdoms with either 1 or 2 BDs *N*-terminal to the ET domain (Supplementary Table S1). This set of sequences contains previously undescribed BET proteins from protists and well-characterized mammalian BET proteins. Phylogenetic analysis of BET proteins revealed a distinct pattern in the number of bromodomains present in different major taxonomic groups (Figure 5). Specifically, plant and protist BET proteins consistently exhibit a single bromodomain, while their fungal and metazoan counterparts display variation, some possessing one or two bromodomains. Also, the distance from the end of the last BD to the start of the ET, typically separated by a primarily disordered region, was measured for each protein (Supplementary Figure S6B). The distance was shorter in protists, plants, and fungi compared to metazoans, with *Tc*BDF3 having the shortest distance. This suggests that mammalian 2BD-ET-type BDFs evolved from a single BD-ET ancestor through the duplication of the BD. This duplication was associated with an increased protein size due to the elongation of the disordered region between the BD and the ET motif.

In conclusion, the affinity of compound A1B4 for *Tc*BDF3 was confirmed by two different methods. In contrast, it did not bind to *Tc*BDF3m, designed to retain its secondary structure while losing its ability to bind to its acetylated ligand, nor the recombinant bromodomain of *Tc*BDF2. In addition, two new compounds related to A1B4 that bind to *Tc*BDF3 were described. Simulations indicate that the compounds can be inserted in the hydrophobic pocket, and they do not support its binding to an alternative domain within *Tc*BDF3. The two new active compounds, identified as 1,3,4-oxadiazoles, demonstrated enhanced trypanocidal activity, particularly against trypomastigotes (the infective stage) and amastigotes (the intracellular stage). Interestingly, prior research by different authors identified 1,3,4-oxadiazoles as inhibitors of human CREBBP bromodomains using a fragment-based high-throughput docking method (Xu et al., 2016). However, our study marks the first instance of this functional group acting as a parasitic bromodomain inhibitor.

An ET domain, which is not reported yet in *Tc*BDF3, has been identified. This domain is also present in *Tc*BDF1, its trypanosomatids orthologs, and various BDFs from protists and plants. These proteins represent highly divergent members of the BET family, where they possess only one bromodomain instead of the typical two found in highly studied BET proteins. Phylogenetic analysis suggests that the 1BD-ET domain architecture was present in ancient eukaryotic cells. On the contrary, the 2BD-ET architecture is associated with more complex organisms such as metazoans. This taxonomic divergence in BD composition suggests evolutionary divergence and potentially distinct functional roles of BET proteins across kingdoms. The fact that 1,3,4-oxadiazoles have previously been described as inhibitors of CREBBP but not BET bromodomains reinforces this idea.

On the other hand, the function of 1BD-ET may also differ among protists. The components of multiprotein nuclear complexes containing BDs in trypanosomatids are only beginning to be identified (Staneva et al., 2021; Jones et al., 2022; Vellmer et al., 2022). The Conserved Regulators of Kinetoplastid Transcription (CRKT) Complex, described in *Leishmania* but also present in *T. brucei*, include the double BD protein BDF5 along with BDF3 and BDF8. In this report, BDF1 and BDF4 were also detected in the proximity proteome of BDF5 (Jones et al., 2022). The unique features of these species result in a “polybromo complex.” Although the CRKT complex is essential, there may be redundancy in the function of bromodomains within the complex, making it more difficult to identify compounds that bind to these BDs with effective antiparasitic activity. In *T. cruzi*, the situation is different. Our group demonstrated that *Tc*BDF1 and *Tc*BDF3 are not nuclear. Furthermore, none were found in proximity labeling experiments using *Tc*BDF5 and *Tc*BDF8 TurboID fusion proteins (Rodríguez Araya, unpublished results). Furthermore, *Tc*BDF1 and *Tc*BDF3 have different cellular localizations than their homologs in *T. brucei* indicating different functions in different trypanosomatids (glycosomal and microtubule-associated, respectively).

The essentiality of these proteins is supported by the phenotypes observed after their overexpression as wild-type and dominant negative mutants, and by the fact that complete knockouts for both *Tcbdf1* and *Tcbdf3* genes could not be achieved using CRISPR/Cas9 genome editing despite several attempts (unpublished results). In this context, *Tc*BDF3 is notable due to its association with the cytoskeleton and a function that is not yet fully understood and does not seem to be shared by any other BD-containing protein. Furthermore, the classification of *Tc*BDF3 as a member of the BET family is significant, given the success of developing BET BD inhibitors to treat cancers and inflammatory conditions (To et al., 2023). Unlike its mammalian counterparts, *Tc*BDF3’s single BD, unique structure, and cytoplasmic localization make it a distinctive target that could be exploited without affecting human BET proteins, thus reducing potential side effects. The success of 1,3,4-oxadiazoles as inhibitors of *Tc*BDF3 further underscores the potential to target this protein in *T. cruzi*. By focusing on atypical BET bromodomains, we can develop highly selective inhibitors that disrupt the parasite’s lifecycle, offering a novel and effective strategy against this neglected tropical disease with limited treatment options.

## Data Availability

We have deposited the raw data in the repository of the National University of Rosario (https://dataverse.unr.edu.ar/dataverse/rda) with the doi: https://doi.org/10.57715/UNR/NWDOCK.

## Footnotes

^1^https://clinicaltrials.gov/, last consultation dated 30 July 2024.
