# Supplementary material for: 1,3,4-oxadiazoles as inhibitors of the atypical member of the BET family bromodomain factor 3 from Trypanosoma cruzi (TcBDF3)
Source: Front Microbiol. 2024 Oct 1;15:1465672. doi: 10.3389/fmicb.2024.1465672 (PMC11473290; doi:10.3389/fmicb.2024.1465672)

Supplementary Material

1,3,4-Oxadiazoles as inhibitors of the atypical member of the BET family Bromodomain Factor 3 from *Trypanosoma cruzi* (*Tc*BDF3)

Victoria Lucia Alonso[1,2], Andrea Escalante[2], Elvio Rodríguez Araya[1,2], Gianfranco Frattini[2,3], Luis Emilio Tavernelli[1#], Diego M. Moreno[2,3], Ricardo Furlan[2], Esteban Serra[1,2*]

[1]Instituto de Biología Molecular y Celular de Rosario, CONICET-UNR. Rosario, Argentina.

[2]Facultad de Ciencias Bioquímicas y Farmacéuticas, Universidad Nacional de Rosario. Rosario, Argentina.

[3]Instituto de Química Rosario, CONICET-UNR. Rosario, Argentina.

#Current address: School of Infection and Immunity. University of Glasgow, Glasgow, Scotland, UK.

1 Supplementary Figures.

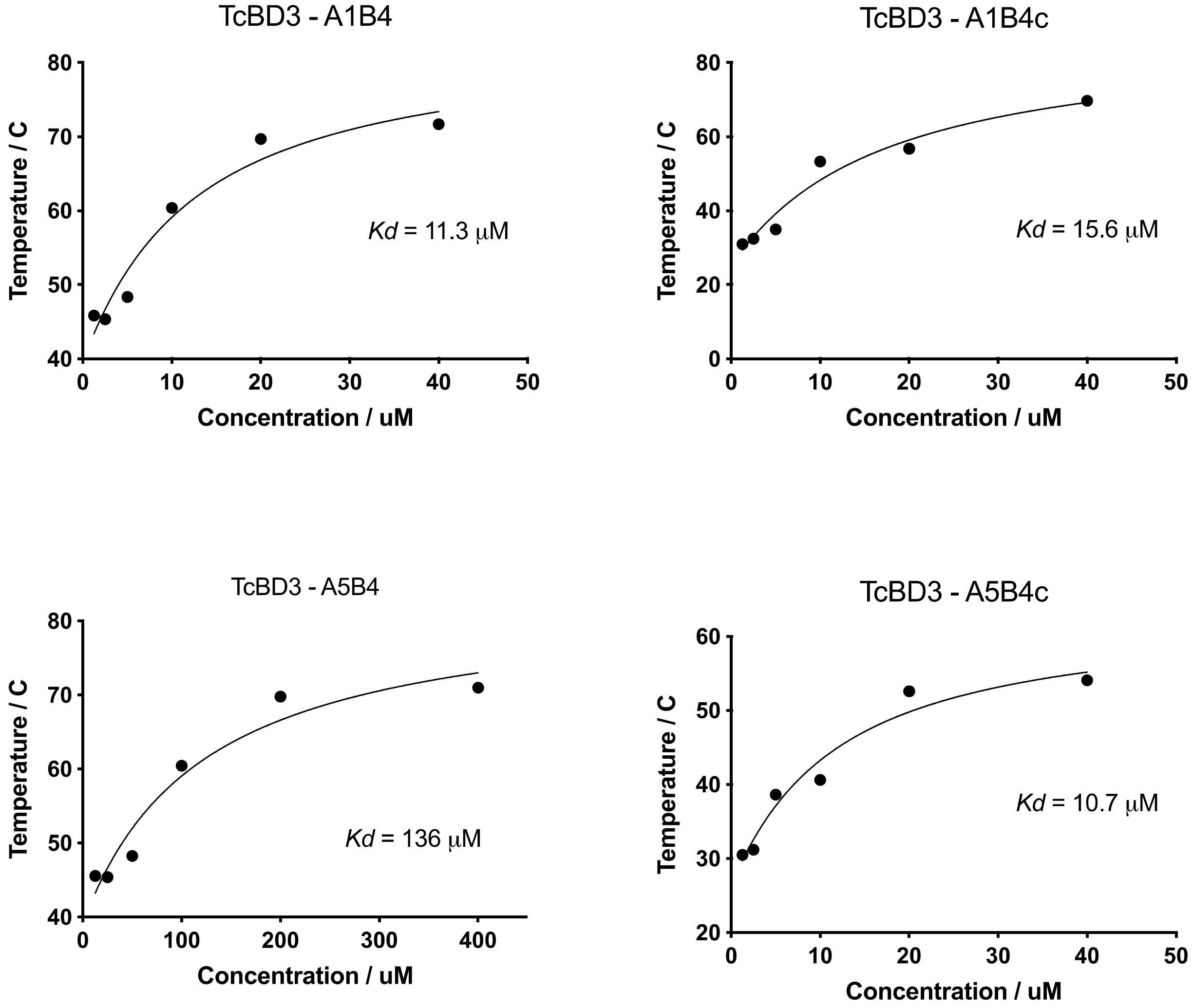

Supplementary Figure S1: Six concentrations of each compound were used from 0.5 to 40 μM, except for A5B4 where we used concentrations ranging from 12.5 to 400 μM. The melting temperatures of *Tc*BD3 with each concentration of compound obtained were used to calculate the *Kd*s. DMSO was used as a control sample. All thermal shift assays were performed at least in triplicate with reproducibility of parameters within ±10%. The change of melting temperature versus the concentration of the ligand concentration was plotted and fitted in GraphPad Prism 9.0 using the DSF single binding site equation.

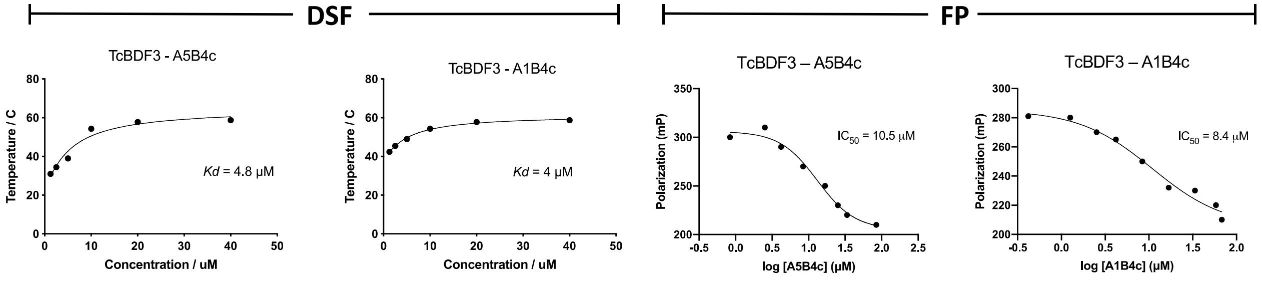

Supplementary Figure S2: For DSF analysis six concentrations of the cyclic compound were used (from 1.25 to 40 μM) and the melting temperatures of *Tc*BDF3 with each concentration of compound obtained were used to calculate the *Kd*s. DMSO was used as a control sample. All thermal shift assays were performed at least in triplicate with reproducibility of parameters within ±10%. The change of melting temperature versus the concentration of the ligand concentration was plotted and fitted in GraphPad Prism 9.0 using the DSF single binding site equation. For FP the cyclic compounds were titrated against a constant concentration of BSP-Alexa488 (0.5 mM) in the presence of 100 μM *Tc*BDF3. Data was analyzed in GraphPad Prism 9.0 by fitting mP data using a sigmoidal dose-response nonlinear regression curve.

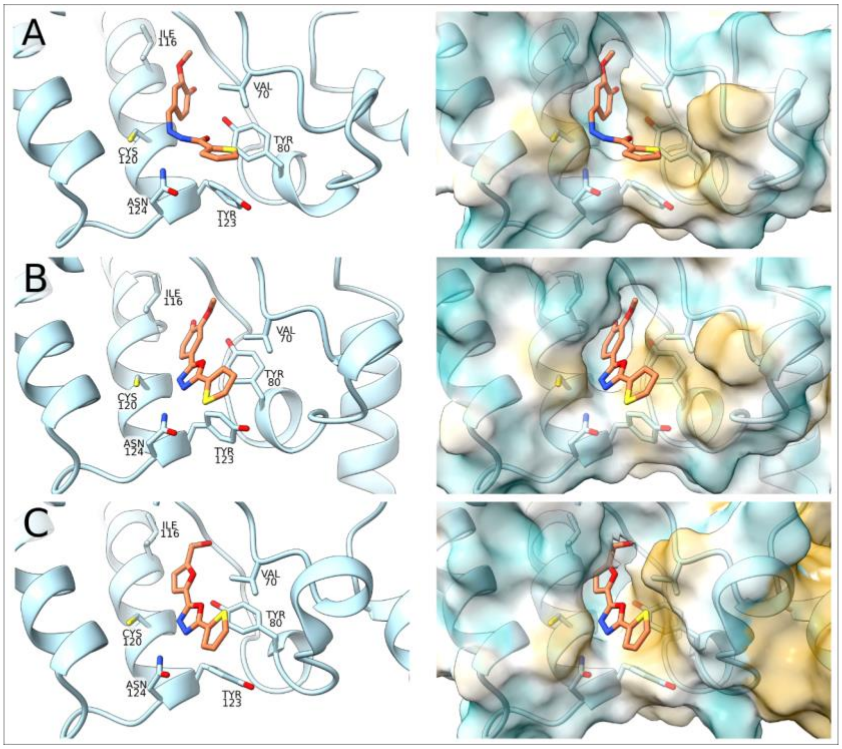

Supplementary Figure S3: *Tc*BDF3-ligand structures obtained from molecular dynamics simulations. In the left panel, the C atoms of the ligands are shown in orange and the C atoms of the *Tc*BDF3 are in light blue. O atoms are colored in red, N atoms in blue, and S atoms in yellow. In the right panel, the *Tc*BDF3 is represented as a surface and the ligands are depicted in liquorice format. **A)** A1B4 **B)** A1B4c **C)** A5B4c.

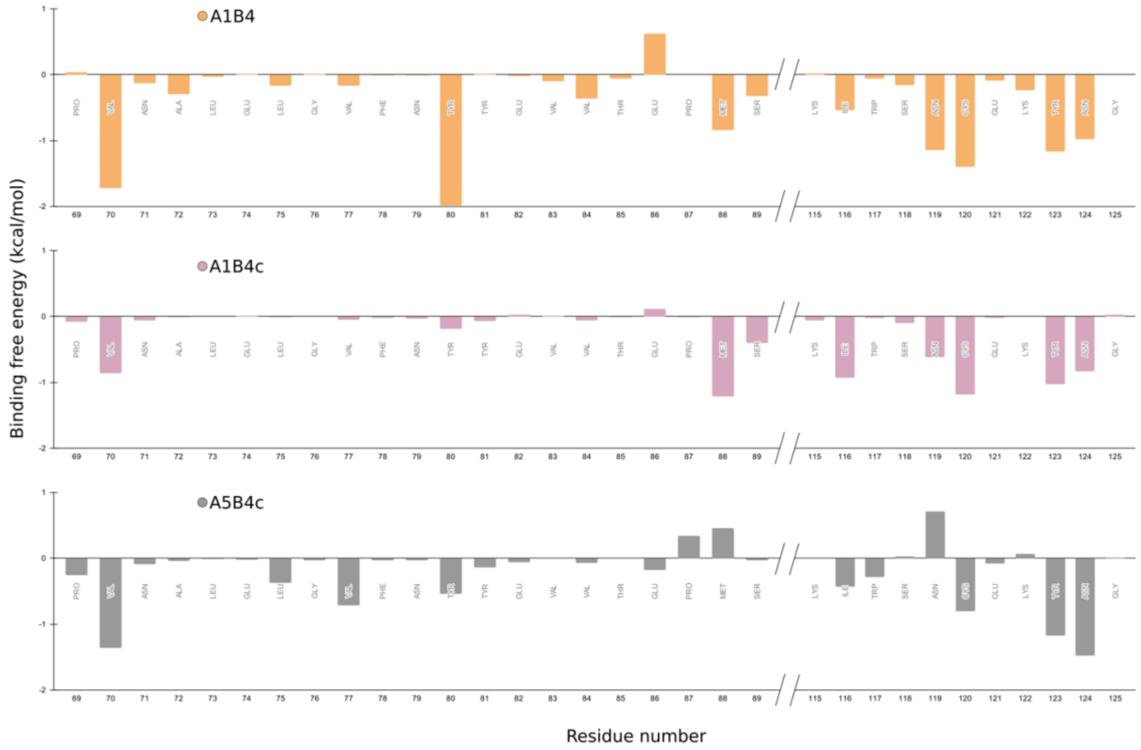

Supplementary Figures S4: Molecular Dynamics simulations. (MM-PBSA). Per-residue binding free energy of the three *Tc*BDF3-ligand complexes. TcBDF3:A1B4 (top plot), TcBDF3:A1B4c (middle plot) and TcBDF3: A5B4c (bottom plot).

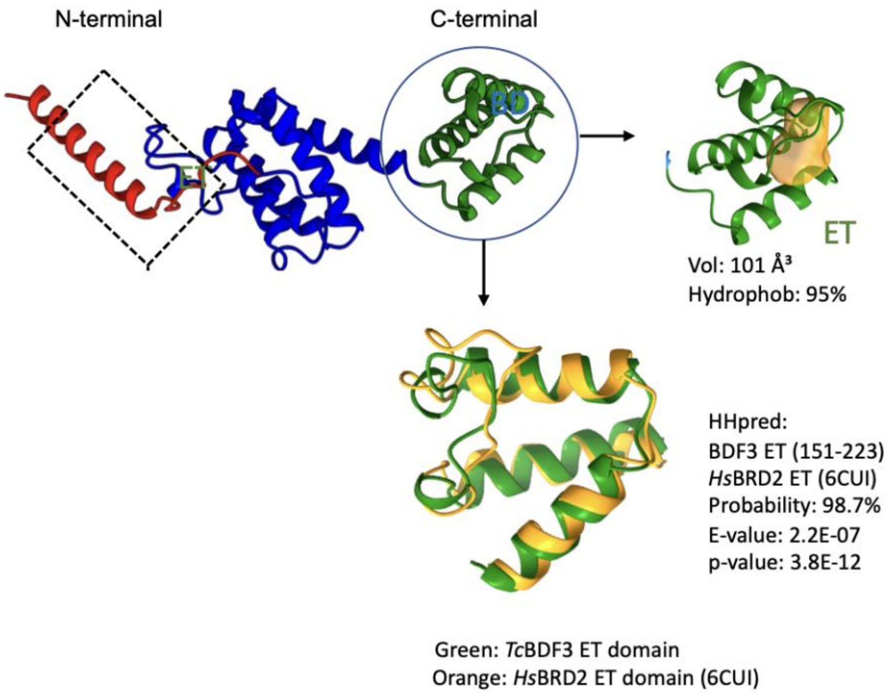

Supplementary Figure S5: *Tc*BDF3 Extra-Terminal domain structural analysis. Volume and hydrophobicity of the ET cavity found in *Tc*BDF3 calculated with CAVIAR. Below is the structural alignment of the *Tc*BDF3 ET domain (amino acids 151 to 223) in green and the ET domain from human BRD2 (Pfam:6CUI) in orange, performed with HHpred.

A

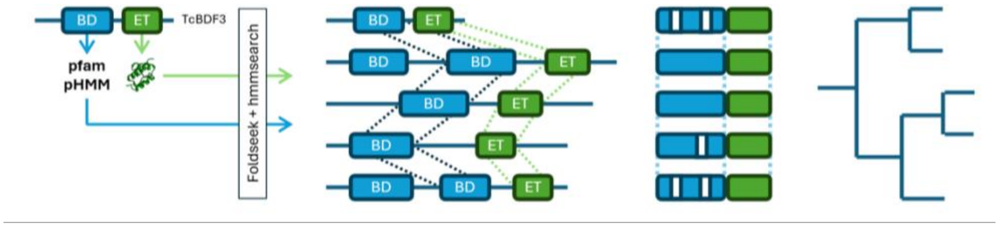

B

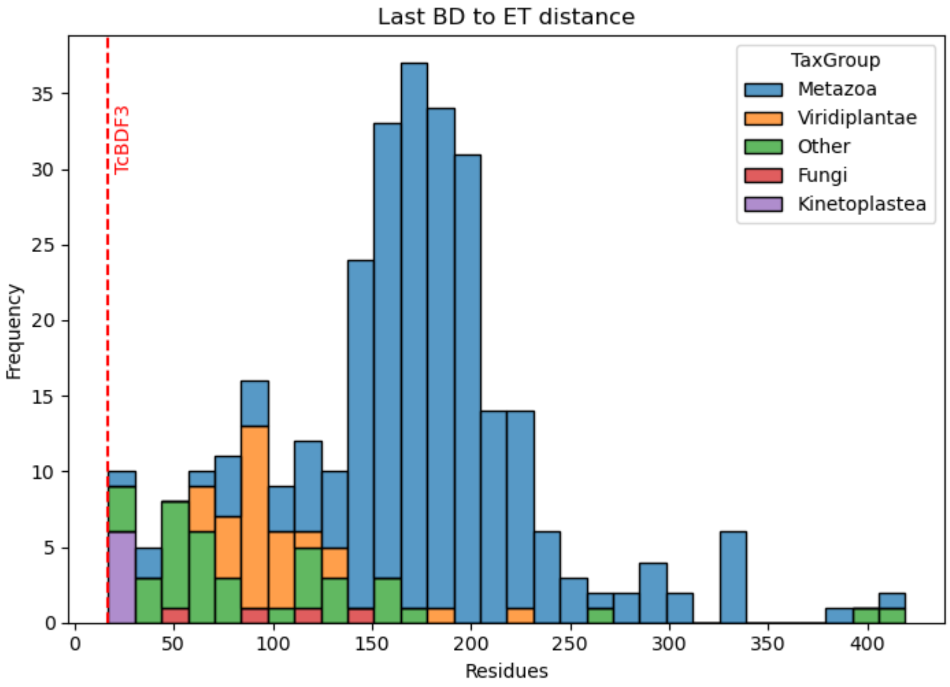

Supplementary Figure S6: BET proteins phylogenetic analysis. **(A)** Schematic representation of the methodology used for the phylogenetic analysis of ET-containing bromodomain factors. **(B)** Distance distribution between the last BD and the ET domain. *Tc*BDF3 has 17 residues between the last amino acid of the BD detected by hmmsearch and the first residue of the ET domain (dashed red line).

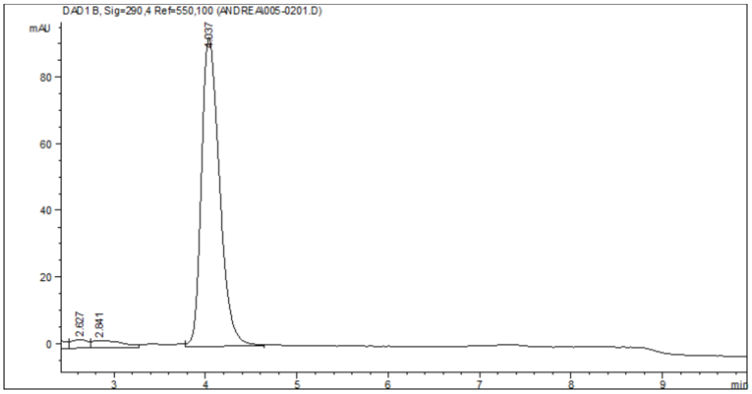

Supplementary Figure S7: HPLC-UV chromatogram of synthesized A1B4c-BOC showed at λ= 290 nm. A solution of A1B4c-boc was injected into a reverse-phase column (Hypersil GOLD C18, 3 µm, 2.1 mm × 150 mm, Thermo Scientific). Elution was carried out at 300 µL/min with a gradient of acetonitrile containing 0.1% formic acid (channel A) and water containing 0.1% formic acid (channel B). Conditions: Column temperature: 30 °C. Injection volume: 2 µL. Elution is given as time (min)/channel A (%): 0-10/40.

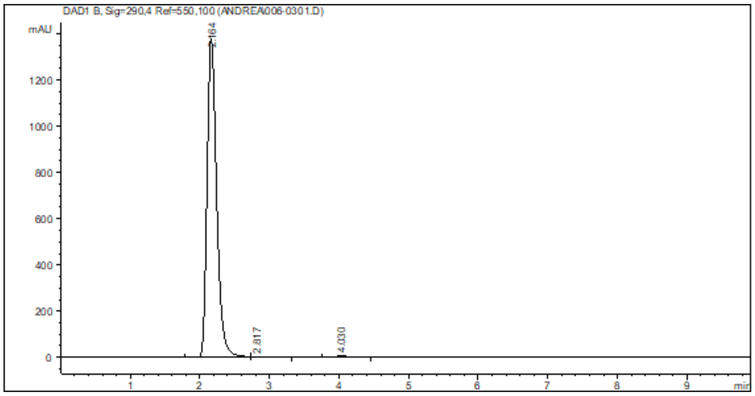

Supplementary Figure S8: LC-UV chromatogram of synthesized A_1B_{4c} showed at λ= 290 nm. A solution of A_1B_4c was injected into a reverse-phase column (Hypersil GOLD C18, 3 µm, 2.1 mm × 150 mm, Thermo Scientific). Elution was carried out at 300 µL/min with a gradient of acetonitrile containing 0.1% formic acid (channel A) and water containing 0.1% formic acid (channel B). Conditions: Column temperature: 30 °C. Injection volume: 2 µL. Elution is given as time (min)/channel A (%): 0-10/40.

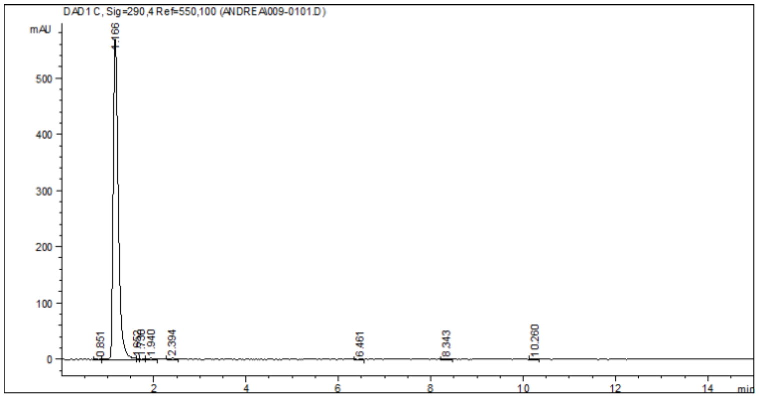

Supplementary Figure S9: HPLC-UV chromatogram of synthesized A5B4c showed at λ= 290 nm. A solution of A5B4c was injected into a reverse-phase column (Hypersil GOLD C18, 3 µm, 2.1 mm × 150 mm, Thermo Scientific). Elution was carried out at 300 µL/min with a gradient of acetonitrile containing 0.1% formic acid (channel A) and water containing 0.1% formic acid (channel B). Conditions: Column temperature: 30 °C. Injection volume: 2 µL. Elution is given as time (min)/channel A (%): 0-15/40

Supplementary Figure S10: NMR spectra and Chromatograms of the synthesized compounds.

^{1}HNMR of *(E,Z)-tert-butyl (2-methoxy-4-((2-(thiophene-2-carbonyl) hydrazono)methyl)phenyl) carbonate*

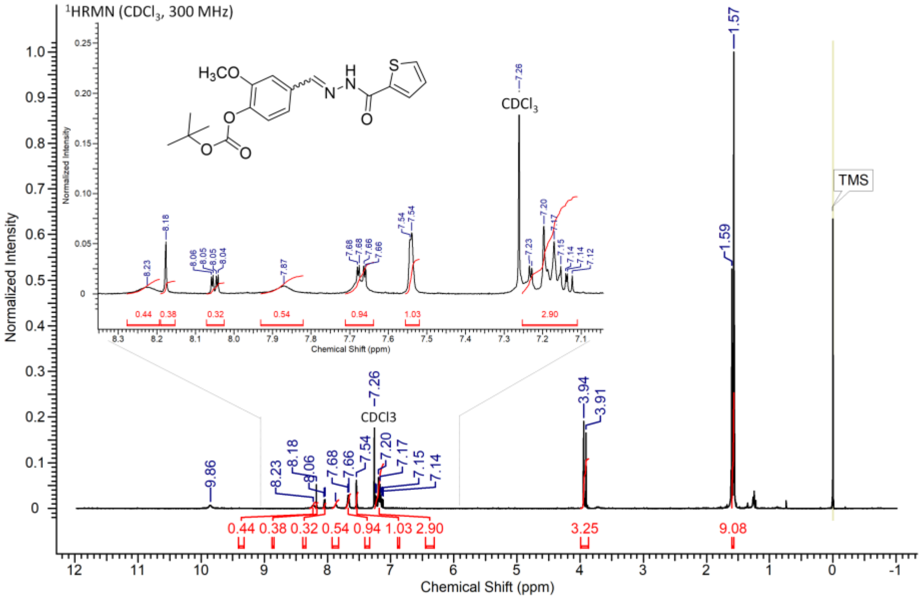

^{1}H and ^{13}C NMR spectra of *tert-butyl (2-methoxy-4-(5-(thiophen-2-yl)-1,3,4-oxadiazol-2-yl)phenyl) carbonate*

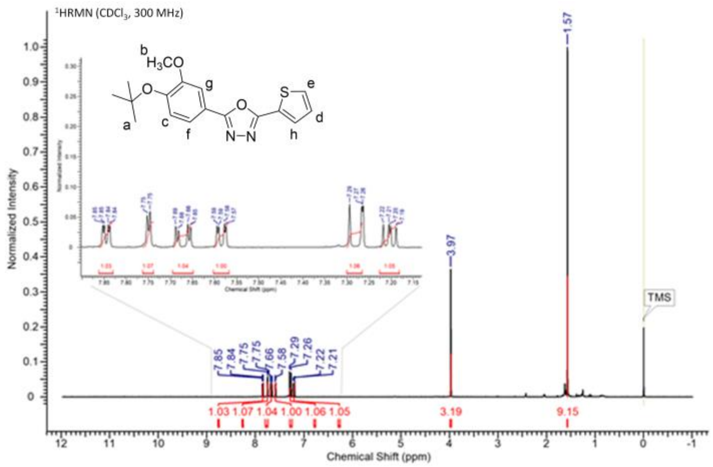

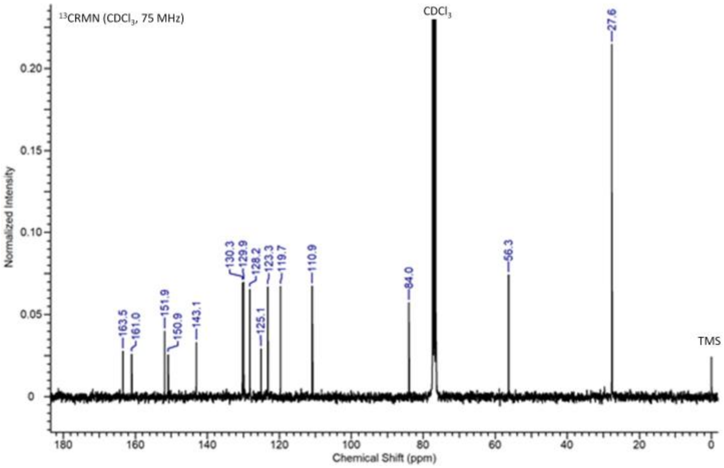

^{1}H and ^{13}C NMR spectra of *2-methoxy-4-(5-(thiophen-2-yl)-1,3,4-oxadiazol-2-yl)phenol* (**A_1B_{4c}**)

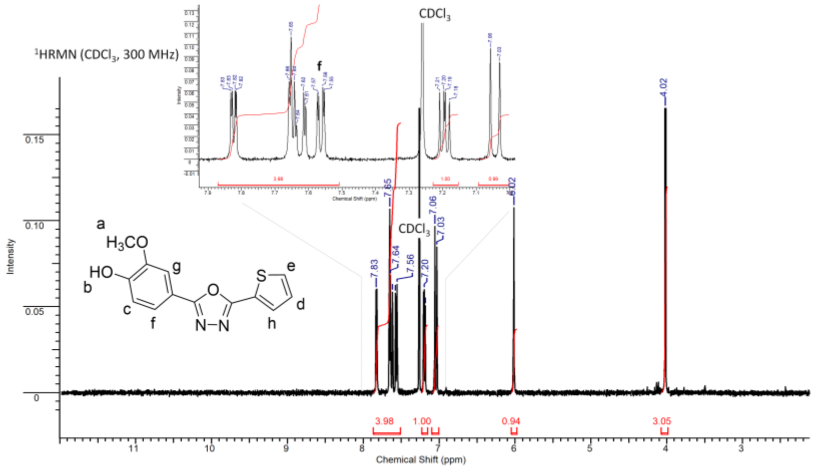

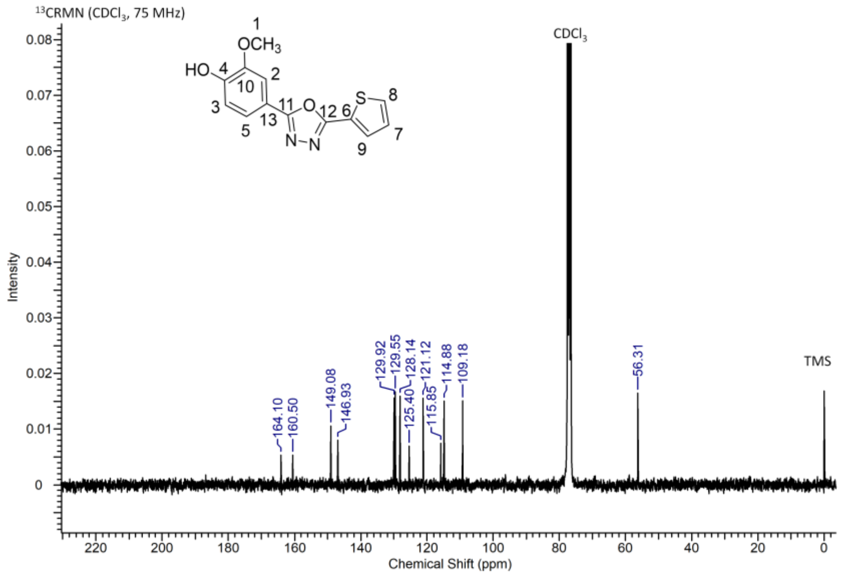

^{1}H and ^{13}C NMR spectra of *(E/Z)-N'-((5-(hydroxymethyl) furan-2-yl) methylene) thiophene-2-carbohydrazide* (**A_5B_4**)

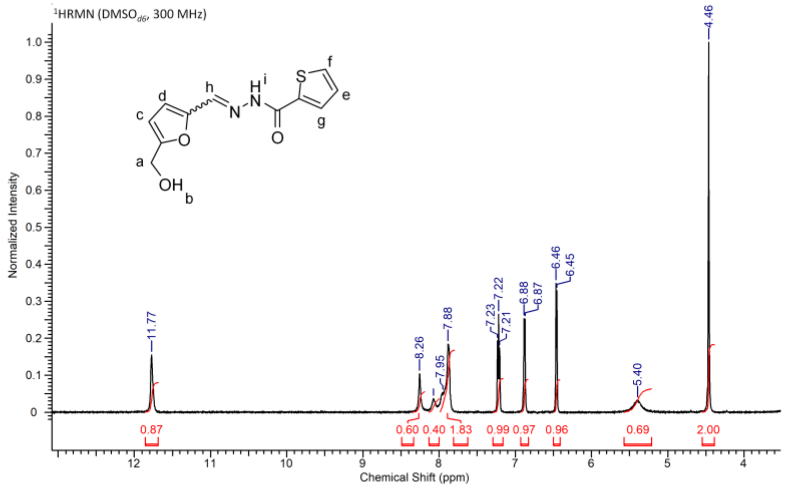

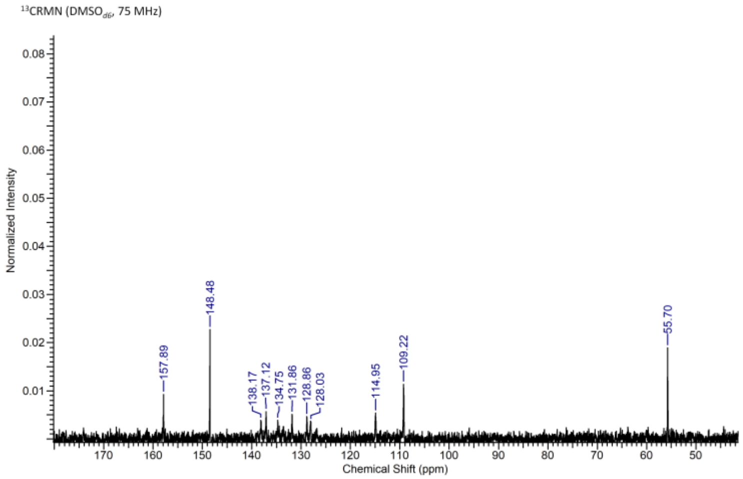

^{1}H and ^{13}C NMR spectra of *(5-(5-(thiophen-2-yl)-1,3,4-oxadiazol-2-yl)furan-2-yl)methanol)* (**A_5B_{4c}**)

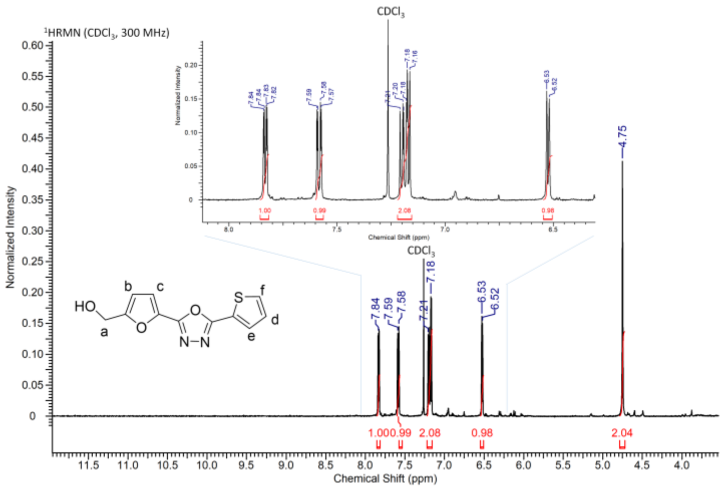

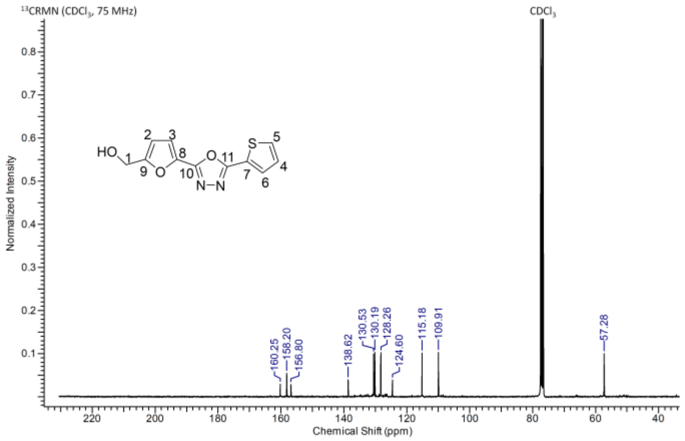

Supplement: Supplementary file 1 [file Data_Sheet_1.PDF]
